# How do SMA-linked mutations of *SMN1* lead to structural/functional deficiency of the SMA protein?

**DOI:** 10.1371/journal.pone.0178519

**Published:** 2017-06-01

**Authors:** Wei Li

**Affiliations:** Medical College, Shantou University, Shantou City, Guangdong Province, China; "INSERM", FRANCE

## Abstract

Spinal muscular atrophy (SMA) is an autosomal recessive neuromuscular disease with dysfunctional α-motor neurons in the anterior horn of the spinal cord. SMA is caused by loss (∼95% of SMA cases) or mutation (∼5% of SMA cases) of the survival motor neuron 1 gene *SMN1*. As the product of *SMN1*, SMN is a component of the SMN complex, and is also involved in the biosynthesis of the small nuclear ribonucleoproteins (snRNPs), which play critical roles in pre-mRNA splicing in the pathogenesis of SMA. To investigate how SMA-linked mutations of *SMN1* lead to structural/functional deficiency of SMN, a set of computational analysis of SMN-related structures were conducted and are described in this article. Of extraordinary interest, the structural analysis highlights three SMN residues (Asp44, Glu134 and Gln136) with SMA-linked missense mutations, which cause disruptions of electrostatic interactions for Asp44, Glu134 and Gln136, and result in three functionally deficient SMA-linked SMN mutants, Asp44Val, Glu134Lys and Gln136Glu. From the computational analysis, it is also possible that SMN’s Lys45 and Asp36 act as two electrostatic clips at the SMN-Gemin2 complex structure interface.

## Introduction

### The spinal muscular atrophy-determining genes

Spinal muscular atrophy (SMA) is an autosomal recessive neuromuscular disease with α-motor neuron dysfunction and muscular atrophy [1]. SMA is caused by loss (∼95% of SMA cases) or mutation (∼5% of SMA cases) of the survival motor neuron gene 1 *SMN1* (telomeric *SMN*, *telSMN* or *SMN1*, GenBank: U18423) [2]. *SMN1* exists in the 5q13 region of the human chromosome, a nearly identical *survival motor neuron 2* gene *SMN2* (centromeric *SMN*, *cenSMN* or *SMN2*, GenBank: NM_022875) also exists in the 5q13 region [2]. The two genes have been extensively characterized and reviewed in detail [1–8].

### The survival motor neuron protein in the pathogenesis of SMA

The survival motor neuron (SMN) protein is the product of the SMA-determining *survival motor neuron gene* *SMN1*, therefore, it is also called the SMA protein. The 38-kD SMN protein locates in the cytoplasm and the nucleus of all cells [9–11]. In the nucleus, SMN (formerly termed Gemin1) is found in dot-like nuclear structures called gems [2, 12].

In the molecular pathogenesis of SMA, of particular interest is an exon 7-skipping splicing defect identified in the pre-mRNA editing of the *SMN2* gene [4]. Due to this splicing defect, *SMN2* predominantly produces exon 7–skipped transcripts, which encodes a truncated isoform of the SMN protein (SMNΔ7 or SMN2 with 282 residues, in comparison with the full-length SMN protein with 294 residues (SMN1 or FL-SMN).

In pre-mRNA editing, spliceosome is the major functional unit, spliceosomal small nuclear ribonucleoproteins (snRNPs) are essential components of the nuclear pre-mRNA processing machinery [13–16]. Here, in SMA, the SMN protein with an A111G mutation (SMNA111G) is capable of snRNP assembly, and can rescue SMA mice that lack *Smn* and contain either one or two copies of *SMN2*. This correction of SMA was directly linked to the correction of snRNA levels and snRNP assembly activity in the spinal cord, which supports snRNP assembly as the critical function affected in SMA and suggests that the levels of snRNPs are critical to motor neurons [17].

In the development of SMA, the SMN protein plays an important role in pre-mRNA processing, because the biogenesis of spliceosomal snRNPs is promoted by the SMN complex [14, 18, 19], within which SMN forms oligomers and directly interacts via its N-terminus with Gemin2 and via its Tudor domain with spliceosomal (Sm) proteins [13, 20, 21]. Moreover, the SMA disease severity was also found to correlate with the oligomerization of SMN, which is required before the formation of SMN complexes [22]. A gel filtration investigation showed that most of the refolded SMN was octameric (∼400 kD) or larger [22, 23], the exact number of SMN monomers in an SMN complex is not known yet, nor is it known whether the oligomerization is dependent on the concentration of the SMN protein [6].

To investigate how SMA-linked mutations of *SMN1* lead to structural/functional deficiency of SMN, a set of computational analysis of SMN-related structures were conducted and are described in detail in the following Methods section.

## Methods

**Fig 1:**
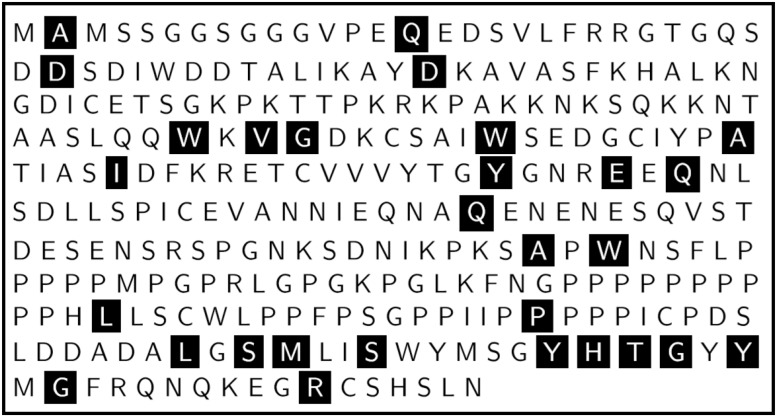
The amino acid sequence and point mutation map of SMN. The amino acid sequence of SMN with SMA-linked *SMN1* point mutations highlighted with white texts and black backgrounds.

SMA-linked *SMN1* mutations (including missense, nonsense, insertion and deletion mutations) exist throughout the SMN protein (Fig 1), and are inextricably linked to the still enigmatic molecular pathogenesis of SMA [6, 24, 25].

### Experimentally determined SMN-related structures

For the computational analysis, the SMN-related structures (Table 1) are retrieved from the wwPDB database [30] with two search parameters (Text Search for: survival motor neuron protein and Molecule: Survival motor neuron protein) available as of May 7, 2017.

**Table 1:** SMN-related structures from the wwPDB database [30]. In this table, TBP (for 4qq6) means to be published, NA (for 4nl6 and 4nl7) means that the structure was from the wrong sample and withdrawn at the request of the author, NumMDL represents the number of structure models in the PDB format file.

PDB ID	Ref	Method	NumMDL	Title of the structure
1g5v	[26]	NMR	10	Solution structure of the Tudor domain of the human SMN protein
1mhn	[26]	Xray	1	High resolution crystal structure of the SMN Tudor domain
2leh	[21]	NMR	32	Solution structure of the core SMN-Gemin2 complex
3s6n	[27]	Xray	1	Crystal structure of the Gemin2-binding domain of SMN,Gemin2 in complex with SMD1/D2/F/E/G from human
4a4e	[28]	NMR	20	Solution structure of SMN Tudor domain in complex with symmetrically dimethylated arginine
4a4g	[28]	NMR	20	Solution structure of SMN Tudor domain in complex with asymmetrically dimethylated arginine
4gli	[29]	Xray	1	Crystal structure of human SMN YG-dimer
4qq6	TBP	Xray	1	Crystal structure of Tudor domain of SMN1 in complex with a small organic molecule
4v98	[14]	Xray	1	Small nuclear ribonucleoprotein Sm D1, Small nuclear ribonucleoprotein Sm D2, Small nuclear ribonucleoprotein E, Small nuclear ribonucleoprotein F, LD23602p, CG10419, Icln, Small nuclear ribonucleoprotein G
4nl6	[13]	Xray	1	NA
4nl7	[13]	Xray	1	NA

### Computational analysis of the SMN-related structures

For the SMN-related structures listed in Table 1, a set of computational analysis was conducted, including salt bridge, hydrogen bond and solvent accessible surface area (SASA) analysis.

First, a simple VMD script was used to split each SMN-related NMR ensemble (multiple-frame PDB file) into a set of single-frame PDB format files. The tcl script is provided as a supplementary file (S3 File).

The salt bridge analysis was conducted with an in-house python script only for titrateable residues (Asp, Glu, Lys, Arg and His), 4.0 Å was used as the cutoff distance for the two oppositely charged groups [31]. The python script is provided as a supplementary file (S4 File).

The hydrogen bond analysis was also conducted for only side chain nuclei with an in-house python script, and employed two geometric criteria: (a) a cutoff value of the angle formed by acceptor (A), donor (D) and hydrogen (H) (∠*ADH*) of 30°; (b) a cutoff value of donor-acceptor distance at 3.0 Å. That is, a hydrogen bond is only considered to be formed if ∠*ADH* is not larger than 30° and the donor-acceptor distance is not larger than 3.0 Å [32]. The python script is provided as a supplementary file (S5 File).

The SASA values were calculated by the DSSP program [32, 33] for all residues in the structurally determined region of the SMN-related structures (Table 1), the intrinsic SASA values used here are the standard SASA values used by NACCESS [34].

## Results

The critical function that is affected in SMA is snRNP assembly, and it is well known that the snRNP biogenesis pathway involves four steps: (A) SMN oligomerization [22], (B) SMN interaction with Gemin proteins [27], (C) SMN binding to the Sm proteins of snRNPs, which are key constituents of spliceosomes [15], (D) SMN-coilin interaction in Cajal bodies [5], the nuclear organelles involved in the maturation of spliceosomal snRNPs.

In the computational analysis here, a residue is defined as potentially important if its side chain is found to be involved in a salt bridge or a hydrogen bond, or its SASA is smaller than 30% of its standard SASA value. These potentially important SMN residues are listed in the first two columns of Table A in S2 File. Among them, only seven residues (Asp44, Trp92, Trp102, Ala111, Ile116, Glu134 and Gln136) were found to be involved in SMA-linked point mutations of *SMN1*, as highlighted with white texts and black backgrounds in Table A in S2 File.

### Asp44 in the Gemin2-binding domain of SMN

Asp44 is in the exon 2a of *SMN1* (the Gemin2-binding domain), and involved in an SMA-linked Asp44Val (D44V) missense mutation [35], which involves a substitution of Asp44’s charged side chain by Val44’s hydrophobic side chain.

Of extraordinary functional significance is that SMN’s Gemin2 binding activity is totally suppressed by the D44V mutation in *SMN1* [27]. Moreover, the D44V SMN mutant (SMND44V)’s snRNP assembly activity is lower than that of the wild-type SMN (FL-SMN or SMN1) [36].

In the snRNP assembly, one important participant is the SMN-Gemin2 complex, whose core structure was determined by solution-state nuclear magnetic resonance (NMR) spectroscopy (PDB ID: 2leh, number of structure models in the NMR ensemble is 32, i.e., NumMDL = 32) [27].

In the computational analysis of the SMN-Gemin2 complex (PDB ID: 2leh), three salt bridges were identified between the buried side chains (Table 2) of two charged residues, i.e., SMN’s Asp44 (B44Asp) and Gemin2’s Arg213 (A213Arg), the distance between the geometric centers of two oppositely charged groups was 3.50 ± 0.23 Å.

**Table 2:** SASA values of SMN’s Asp44 and Gemin2’s Arg213 (PDB ID: 2leh) [21]. In this table, SASA-Mean, SASA-Std, SASA-Intrinsic and SASA-Ratio represent for SMN’s Asp44 and Gemin2’s Arg213 the average SASA value, one standard deviation from the average SASA value, the intrinsic SASA value [34], and the ratio of SASA-Mean divided by SASA-Intrinsic, respectively.

Residue	SASA-Mean (Å^2^)	SASA-Std (Å^2^)	SASA-Intrinsic (Å^2^)	SASA-Ratio
A213Arg	130.41	19.16	238.76	0.55
B44Asp	101.12	9.16	140.39	0.72

Specifically, for the side chain charged groups of SMN’s Asp44 and Gemin2’s Arg213, the distance distribution for all 32 NMR structure models (PDB ID: 2leh) is 8.33 ± 2.55 Å for the O_δ2_-N_η2_ nucleus pair, and 8.71 ± 1.82 Å for the O_δ2_-N_η1_ nucleus pair, and 8.08 ± 3.02 Å for the O_δ1_-N_η2_ nucleus pair, and 8.59 ± 2.27 Å for the O_δ1_-N_η1_ nucleus pair. These distance distributions argue against the existence of the Asp44-Arg213 salt bride, although three were computationally identified in the NMR ensemble (PDB ID: 2leh, NumMDL = 32).

Yet, in an subsequent analysis of the experimental NMR restraints deposited in the BMRB database (PDB ID: 2leh, BMRB ID: 17711) [21], a set of chemical shift assignments were found to be missing for a group of crucial side chain nuclei, including C_γ_ of SMN’s Asp44 and C_ζ_, all side chain nitrogen nuclei and all nitrogen-bonded side chain hydrogen nuclei of Gemin2’s Arg213. The missing assignments of those chemical shifts increases the degree of geometric freedom for the side chains in the experimental structure determation, and result in inadequate accuracy of the geometric definition of the Asp44’s and Arg213’s side chains in the NMR ensemble (PDB ID: 2leh).

Taken together, it is conceivable that the buried side chains of SMN’s Asp44 and Gemin2’s Arg213 form a salt bridge, which constitutes a favourable electrostatic energy contribution to the SMN-Gemin2 complex structural stability [27], and highlights the functionally indispensable roles of the two residues’ charged side chains, considering the experimental observation that the SMN-Gemin2 binding is abrogated by the D44V mutation [27], resulting in a functionally deficient SMA-linked D44V SMN mutant (SMND44V).

In a comparison, Lys41 is a positively charged residue and also a neighbouring residue of Asp44. Functionally different to the SMA-linked D44V mutation, a Lys41Ala (K41A) mutation (not SMA-linked) does not affect SMN-Gemin2 binding [27]. This experimental observation is consistent with the salt bridge analysis of the SMN-Gemin2 complex (PDB ID: 2leh, NumMDL = 32), where 15 salt bridges were identified between the side chains of SMN’s Asp35 and Lys41, with the distance between two oppositely charged groups being 3.37 ± 0.39 Å, and of particular relevance, in the SMN-Gemin2 complex (PDB ID: 2leh), SMN’s Lys41 only forms side bridges with SMN’s Asp35, i.e., the 15 Lys41-Asp35 salt bridges are invariably intramolecular, i.e., within the apo SMN protein, instead of intermolecular, i.e., at the SMN-Gemin2 complex structure interface.

### SMN’s Lys45 and Asp36 at the SMN-Gemin2 interface

**Fig 2:**
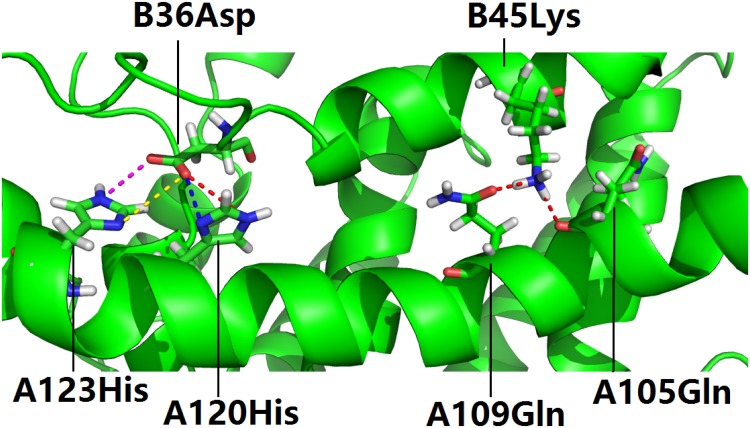
Asp36 and Lys45 at the binding interface of the SMN-Gemin2 complex. In this figure, salt bridges are colored differently as sticks on the left side, and hydrogen bonds as red sticks on the right side, each residue is identified with the chain ID (A for Gemin2 and B for SMN), residue number and three-letter residue code, such as B45Lys. The details of the salt bridges and hydrogen bonds are included in Tables 3 and 4.

At the SMN–Gemin2 complex structure interface (PDB ID: 2leh), SMN’s Asp36 is directed towards the Gemin2 interface, allowing it to interact with Gemin2’s His120 [21]. In the computational analysis of the SMN-Gemin2 complex [27], four salt bridges were identified between the side chains of SMN’s Asp36 (B36Asp) and those of Gemin2’s His120 (A120His) and His123 (A123His), and four hydrogen bonds were found to be formed between SMN’s Lys45 (B45Lys) and Gemin2’s Gln109 (A109Gln), and between B45Lys and Gemin2’s Gln105 (A105Gln), and between SMN’s Asp36 (B36Asp) and Gemin’s Trp124 (A124Trp) in the NMR ensemble of the SMN-Gemin2 complex (PDB ID: 2leh) (Fig 2) [21].

**Table 3:** Two salt bridge pairs formed between A123His and B36Asp and B36Asp and A120His in the fifteenth structure model of the NMR ensemble (PDB ID: 2leh) of the SMN-Gemin2 complex [21]. In the first column, SB represents salt bridge, Inter-atomic distance includes both the distance between the two oppositely charged groups and the distribution of the distances between the two oppositely charged groups for the 32 NMR structure models in the SMN-Gemin2 complex. Color represents the color of the dash lines used to represent the salt bridges in Fig 2 in the main file. In the residue naming scheme here, such as B36Asp, B represents the chain ID used in the PDB file (PDB ID: 2leh), 36 represents the residue number, Asp represents the three-letter code for the amino acid.

SB	Atom A (Residue)	Atom B (Residue)	Inter-atomic distance (Å)	Color
SB1	NE2 (A123His)	OD2 (B36Asp)	3.1 (5.73 ± 0.93)	Magenta
SB2	ND1 (A123His)	OD1 (B36Asp)	4.0 (7.04 ± 1.01)	Yellow
SB3	ND1 (A120His)	OD1 (B36Asp)	2.6 (6.87 ± 0.94)	Blue
SB4	NE2 (A120His)	OD1 (B36Asp)	3.7 (5.57 ± 0.64)	Red

**Table 4:** Four hydrogen bonds formed between B45Lys and A109Gln and B45Lys and A105Gln of the NMR ensemble of the SMN-Gemin2 complex (PDB ID: 2leh) [21]. Apart from those mentioned in this table, no more hydrogen bond or salt bridge was found for B45Lys. In the first row, HB represents hydrogen bond, *D* − *A* and *H* − *A* represent the distance between donor and acceptor and hydrogen and acceptor, respectively. HB1, HB2, HB3 and HB4 represent hydrogen bonds that were computationally identified from the eighth, the eighth, the nineteenth and the eleventh structure models of the SMN-Gemin2 complex (PDB ID: 2leh, NumMDL = 32). The unshaded rows represent the values of *D* − *A*, *H* − *A* and ∠*ADH* for the hydrogen bonds. The shaded rows represent the the distributions of *D* − *A*, *H* − *A* and ∠*ADH* for the 32 NMR structure models in the SMN-Gemin2 complex, i.e., the mean ± one standard deviation of the distances or the ∠*ADH* angles for the hydrogen bonds. The residue naming scheme is the same as that used in Table 3.

HB	Acceptor (A)	Donor (D)	Hydrogen (H)	D-A (Å)	H-A (Å)	∠*ADH*(°)
HB1	OE1, A109Gln	NZ, B45Lys	HZ1, B45Lys	2.63	1.68	18.83
HB1	OE1, A109Gln	NZ, B45Lys	HZ1, B45Lys	4.11 ± 1.30	4.13 ± 1.41	86.43 ± 41.56
HB2	O, A105Gln	NZ, B45Lys	HZ3, B45Lys	2.70	1.68	9.22
HB2	O, A105Gln	NZ, B45Lys	HZ3, B45Lys	3.73 ± 0.97	3.76 ± 1.28	84.34 ± 41.27
HB3	O, A105Gln	NZ, B45Lys	HZ2, B45Lys	2.94	2.06	26.72
HB3	O, A105Gln	NZ, B45Lys	HZ2, B45Lys	3.73 ± 0.97	3.64 ± 1.18	76.15 ± 33.94
HB4	OD2, B36Asp	NE1, A124Trp	HE1, A124Trp	2.76	1.95	28.37
HB4	OD2, B36Asp	NE1, A124Trp	HE1, A124Trp	5.91 ± 1.15	5.53 ± 1.19	62.29 ± 10.49

In a visual inspection of Fig 2, the side chains of SMN’s Lys45 and Asp36 are buried in the groove of the SMN-Gemin2 interface, as also supported by the quantitative analysis in Table 5 of the SASA values of SMN’s Lys45 and Asp36 (PDB ID: 2leh) [21].

**Table 5:** SASA values of SMN’s Lys45 and Asp36 (PDB ID: 2leh) [21]. In this table, SASA-Mean, SASA-Std, SASA-Intrinsic and SASA-Ratio represent for SMN’s Lys45 and Asp36 the average SASA value, one standard deviation from the average SASA value, the intrinsic SASA value [34], and the ratio of SASA-Mean divided by SASA-Intrinsic, respectively.

Residue	SASA-Mean (Å^2^)	SASA-Std (Å^2^)	SASA-Intrinsic (Å^2^)	SASA-Ratio
B45Lys	80.56	13.12	200.81	0.40
B36Asp	28.44	13.25	140.39	0.20

For the salt bridges and the hydrogen bonds described in Fig 2 and Tables 3 and 4, only one/two NMR structure model(s) was/were identified for whole NMR ensemble (PDB ID: 2leh, Num = 32) [21], which presents a strong evidence against the existence of the computationally identified electrostatic interactions described in Fig 2.

Yet, in an analysis of the experimental NMR restraints deposited in BMRB database (PDB ID: 2leh, BMRB ID: 17711) [21], a set of chemical shift assignments were found to be missing for a group of crucial side chain nuclei, including C_γ_ of SMN’s Asp36 and all four nuclei of the side chain NH_3_ group of SMN’s Lys45, the two C_δ_ nuclei of Gemin2’s Gln109 and Gln105, and C_γ_, H_δ1_ and H_ε2_ of Gemin2’s His120, and C_γ_, C_δ2_, H_δ1_ and H_ε2_ of Gemin2’s His123, C_ζ3_, C_δ2_, H_ε3_, H_ζ3_, C_γ_, C_ε2_ and C_ε3_ of Gemin2’s Trp124. The missing assignments of those chemical shifts increases the degree of geometric freedom for the side chains in the experimental structure determation, and result in inadequate accuracy of the geometric definition of the two residues’s side chains which are instrumental in the electrostatic interactions shown in Fig 2.

Taken together, it is reasonable to not rule out the possibility that the deeply buried side chains of SMN’s Lys45 and Asp36 act as two electrostatic clips at the SMN-Gemin2 interface via interactions with Gemin2’s Gln105, Gln109, His120, His123 and Trp124, and that, similar to SMN’s Asp44 and Gemin2’s Arg213, the two SMN residues (Lys45 and Asp36) play stabilizing roles in the SMN-Gemin2 complex structure formation, suggesting that it could be merely a matter of chance in the evolution of the *SMN1* gene that no SMA-linked mutations of SMN’s Lys45 and Asp36 have been identified in SMA patients yet, in light of the experimentally observed SMA-linked missense mutation of D44V in the *SMN1* gene [35].

### Four deeply buried hydrophobic SMN residues

Trp92, Trp102, Ala111 and Ile116 are four hydrophobic SMN residues with SMA-linked mutations in the SMN Tudor domain. With SASA values smaller than 30% of their standard values, Trp92 (12±2%), Trp102 (10±3%), Ala111 (<1%) and Ile116 (14±0.5%) are defined here as deeply buried hydrophobic SMN residues. Furthermore, for the four residues, no salt bridge or hydrogen bond was identified in the computational analysis of the SMN-related structures.

The W92S substitution reduced the binding of the Tudor domain to Sm-B protein by ∼80% [37]. Moreover, in a protein expression study, the mutant W92S SMN protein was expressed at lower levels than the wild-type SMN protein, but no difference was observed in the transcript levels, suggesting the instability of the SMN protein due to the W92S mutation [38].

Trp102 is involved in a nonsense SMA-linked mutation (Trp102X), a premature termination mutations in *SMN1* exon 3, which was identified in two SMA patients with a relatively mild phenotype who had two copies of *SMN2* [39, 40]. More functional study is required for this SMA-linked mutation.

Another hydrophobic residue in the SMN Tudor domain, Ala111 is almost completely buried in the SMN Tudor domain (with an *SASA* value of 0.3 ± 0.48 Å^2^ compared with its standard *SASA* value at 107.9 Å^2^). The Ala111Gly (A111G) mutation [17, 41] reduces, but does not totally suppress, the binding of SMN to Sm proteins, confirming Tudor domain‘s role in SMN‘s binding to Sm proteins [15, 16].

For the A111G mutation, the net structural impact is the removal of the hydrophobic side chain of Ala111 (similar to the A2G mutation). Moreover, as discussed above, the SMNA111G mutant is still capable of snRNP assembly and associating with full-length SMN to form an oligomer in vitro [17]. Collectively, it is conceivable that A111’s deeply buried hydrophobic side chain points towards the interior of the SMN Tudor domain structure and provides a favourable energy contribution to the Tudor domain stability, this intra-domain impact might help explain the reduced (but not abrogated) binding of SMN to Sm proteins.

Ile116 is involved in an SMA-linked Ile116Phe (I116F) missense mutation which was first reported in Spanish patients [24, 25]. The Sm core assembly activity of I116F SMN mutant was found to be significantly lower than that of SMN1 [42].

Taken together, the four SMA-linked mutations highlight the potential significance of the deeply buried hydrophobic side chains of Trp92, Trp102, Ala111 and Ile116 in the SMN Tudor domain. More comprehensive structural and functional research is required to characterize the functional roles of these residues in SMN and also the development of SMA.

### Glu134 and Gln136 in the SMN Tudor domain

Glu134 and Gln136 are involved in two SMA-linked mutations in the SMN Tudor domain, i.e., Glu134Lys (E134K) and Gln136Glu (Q136E), respectively.

Both SMN’s Glu134 and Gln136 are involved in hydrogen bonds with SMN’s Tyr127, as revealed by both the structures of apo SMN Tudor domain [26, 43] and the SMN Tudor domain bound to symmetric and asymmetric dimethylated arginine (sDMA and aDMA) [28], and also by the computational analysis here, where Gln136 was found to form 14 hydrogen bonds with Tyr127 (PDB ID: 4a4g, NumMDL = 20), and Glu134 was found to form 20 (PDB ID: 4a4e, NumMDL = 20) and 19 (PDB ID: 4a4g, NumMDL = 20) hydrogen bonds with Tyr127. In addition, Glu134 also formed 18 (PDB ID: 4a4e, NumMDL = 20) and 12 (PDB ID: 4a4g, NumMDL = 20) hydrogen bonds with SMN’s Ser103.

In the NMR structure of SMN Tudor domain bound to sDMA [28], the E134K mutation reduced binding affinity for sDMA by an order of magnitude. In addition, the Sm core assembly activity of both SMN mutants (SMNE134K and SMNQ136E) were found to be significantly lower than the wild-type SMN [42].

Regardless of whether new salt bridges formed or not due to the two SMA-linked substitutions E134K and Q136E), one approach to understand the above-described functional deficiency of SMN is to structurally examine the two mutations’ consequence(s) at residue positions 134 and 136 in SMN. In the computational structural analysis, local electrostatic interaction disruptions in SMN Tudor domain do arise due to the disruptions of the Glu134- and Gln136-involved hydrogen bond interactions.

For instance, Glu134 provides its O_ε1_ or O_ε2_ as the hydrogen acceptor in the hydrogen bonds, where the two oxygens carry partial negative charges, and the hydrogen bond donor carries partial positive charge. An E134K substitution removes the negatively charged side chain group of Glu134, and installs a positively charged lysine side chain instead. This reversal in the side chain electric charge causes a shift of electrostatic attraction (the hydrogen bond interactions between Glu134 and Tyr127, and between Glu134 and Ser103) to electrostatic repulsion between positively charged Lys134 side chain and the hydrogen bond donor which carries partial positive charge, resulting in local electrostatic interaction disruption in the SMN Tudor domain structure.

Similarly, Gln136 provides its side chain NH_2_ group as the hydrogen donor group in the hydrogen bonds, where the hydrogen carries partial positive charge due to its low electronegativity compared with the directly bonded nitrogen, and the hydrogen bond acceptor carries partial negative charge. A Q136E substitution removes the neutral side chain group of Gln136, along with the hydrogen with partial positive charge in the side chain NH_2_ group of Gln136, and installs a negatively charged glutamate side chain instead. This reversal in the side chain electric charge causes a shift of electrostatic attraction (the hydrogen bond interaction between Gln136 and Tyr127) to electrostatic repulsion between negatively charged Glu136 side chain and the hydrogen bond acceptor which carries partial negative charge, resulting in local electrostatic interaction disruption in the SMN Tudor domain structure.

Overall, the structural consequences of the E134K and the Q136E mutations consist qualitatively of two reversals in the signs of the electrostatic interactions (from attractions to repulsions) involving the two SMN residues at positions 134 and 136. As a result, the two SMA-linked mutations in the Tudor domain constitute two local electrostatically destabilizing sites, contributing to the structural instability of the SMN Tudor domain (the essential part of SMN for the Sm protein-binding), which can help explain the reduced Sm core assembly activity of the two SMA-linked SMNE134K and SMNQ136E mutants.

## Conclusion

**Fig 3:**
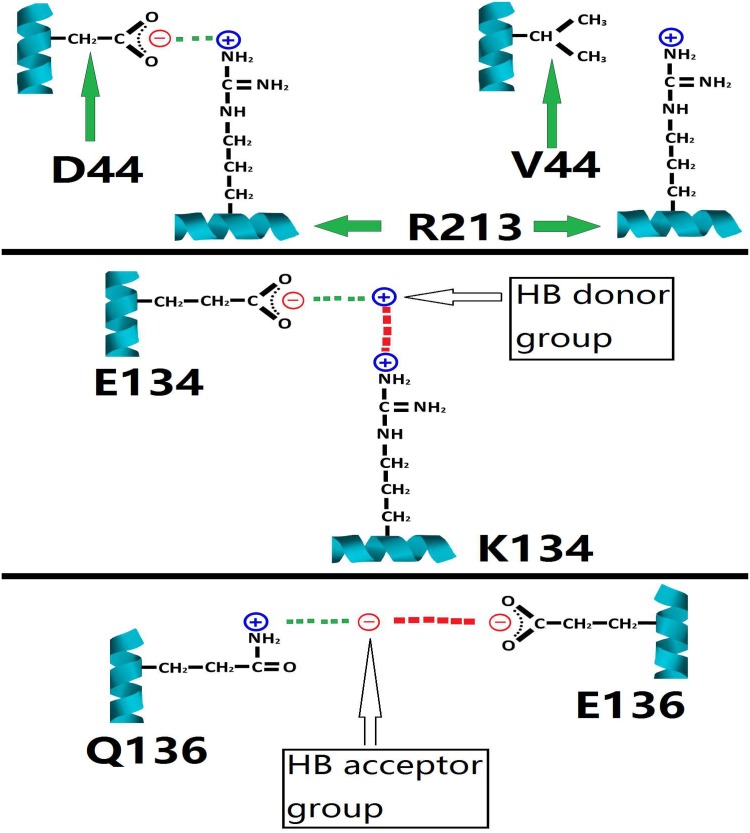
Disruptions of local electrostatic interactions by three SMA-linked missense mutations. In this figure, the electrostatic interaction disruptions for D44V, E134K and Q136E are illustrated in the upper, the middle and the lower panels, respectively. In the three panels, the negative (partial, for hydrogen bond acceptor group) charge is represented with a red circle with a minus sign inside, the positive (partial, for hydrogen bond donor group) charge is represented with a blue circle with a plus sign inside, the salt bridge/the hydrogen bond is represented with a set of green squares, representing electrostatic attraction. For the upper panel, the impact of the mutation is represented with the disappearance of the green squares on the right side, representing the disappearance of the Asp44-Arg213 salt bridge. For the middle and lower panels, the impacts of the mutations are represented with a set of red squares, representing electrostatic repulsion.

To characterize the relationship between SMA-linked mutations of *SMN1* and SMN’s structure and function, a set of computational analysis of SMN-related structures was conducted and described above. With the structural analysis, this article highlights three residues of SMN (Asp44, Glu134 and Gln136), and the electrostatic basis (Fig 3) of how the SMA-linked missense mutations of the three residues cause structural/functional deficiency of SMN, and also a possibility of SMN’s Lys45 and Asp36 acting as two electrostatically stabilizing clips at the SMN-Gemin2 complex structure interface.

In addition, the structural analysis here also suggested potential functional significance of four deeply buried hydrophobic residues (Trp92, Trp102, Ala111 and Ile116) with SMA-linked point mutations in the SMN Tudor domain.

## Discussion

Since the SMN protein (also called the SMA protein) has an established role in snRNP biogenesis and the development of SMA, SMA Patient-derived SMN mutations offer a valuable resource to help characterize the function of SMN. While the functional impact was studied for certain SMA-linked mutations, due to the lack of experimentally determined three-dimensional structure, a structural analysis is not possible yet for those SMN residues with SMA-linked mutations.

For example, in zebra-fish, a Gly264Asp (G264D) missense mutation is linked to presynaptic neuromuscular junction defect via the synaptic vesicle protein SV2 [44]. Interestingly, the G264 of zebra-fish aligns with G279 of human in the amino acid sequence alignment [44]. Moreover, G279 locates in the truncated part of SMNΔ7 compared with FL-SMN, and two SMA-linked missense mutations (G279C and G279V) were also identified for this glycine [3, 42], highlighting the functional significance of Gly279 (and also the truncated C-terminal part) of FL-SMN.

As of May 7, 2017, the wwPDB website [30] produced nine experimentally determined SMN-related structures (Table 1). In terms of amino acid sequence, those SMN-related structures are only SMN fragments, ranging from Gly26 to Lys51, and from Asn84 to Glu147 (S1 File). In between, there is still a structurally not-determined-yet region consisting of 204 SMN residues, calling for continued structural and functional characterization of the SMA protein.

## Supporting information

S1 File Results of the computational analysis of SMN-related NMR structures. This supplementary file (S1_File.pdf) provides the results of the computational analysis of salt bridge, hydrogen bond and solvent accessible surface area (SASA) for four SMN-related structures determined by solution-state nuclear magnetic resonance (NMR) spectroscopy.
(PDF)

S2 File Interpretation and visualization of the results of the computational analysis of SMN-related structures. This supplementary file (S2_File.pdf) presents four supporting figures for the visualization of the salt bridge and the hydrogen bond analysis, a table of computationally identified SMN residues aligned against experimentally identified SMA-linked SMN mutations, and a set of tables describing the chemical shift assignment analysis for the SMN-related SMN structures.
(PDF)

S3 File A tcl script for the splitting of SMN-related NMR ensemble into single-frame PDB files. This tcl script (S3 File) is used in VMD to split NMR ensembles.
(TCL)

S4 File A python script for structure-based computational salt bridge analysis. This supplementary file (S4_File.py) presents a python script for structure-based computational salt bridge analysis. The salt bridge analysis was conducted only for titrateable residues (Asp, Glu, Lys, Arg and His), 4.0 Å was used as the cutoff distance for the two oppositely charged groups.
(PY) Click here for additional data file.

S5 File A python script for structure-based computational hydrogen bond analysis. This supplementary file (S5_File.py) presents a python script for structure-based computational hydrogen bond analysis. The hydrogen bond analysis employs two geometric criteria: (a) a cutoff value of the angle formed by acceptor (A), donor (D) and hydrogen (H) (∠*ADH*) of 30°; (b) a cutoff value of donor-acceptor distance at 3.0 Å.
(PY) Click here for additional data file.
