# Supplementary material for: How do SMA-linked mutations of SMN1 lead to structural/functional deficiency of the SMA protein?
Source: PLoS One. 2017 Jun 1;12(6):e0178519. doi: 10.1371/journal.pone.0178519 (PMC5453535; doi:10.1371/journal.pone.0178519)
Supplement: S1 File — This supplementary file (S1_File.pdf) provides the results of the computational analysis of salt bridge, hydrogen bond and solvent accessible surface area (SASA) for four SMN-related structures determined by solution-state nuclear magnetic resonance (NMR) spectroscopy. (PDF) [file pone.0178519.s001.pdf]

Res	SMN(aa)		A	Salt bridge analysis				Hydrogen bond analysis				SASA analysis(1g5v)($Å^2$)				SASA analysis(2leh)($Å^2$)				SASA analysis(4a4e)($Å^2$)				SASA analysis(4a4g)($Å^2$)			
No.	1	2	A	1	2	3	4	1	2	3	4	Mean	Std	Norm	Ratio	Mean	Std	Norm	Ratio	Mean	Std	Norm	Ratio	Mean	Std	Norm	Ratio
1	M	M	T	0	0	0	0	0	0	0	0	0	0	0	0	0	0	0	0	0	0	0	0	0	0	0	0
2	A	A	T	0	0	0	0	0	0	0	0	0	0	0	0	0	0	0	0	0	0	0	0	0	0	0	0
3	M	M	T	0	0	0	0	0	0	0	0	0	0	0	0	0	0	0	0	0	0	0	0	0	0	0	0
4	S	S	T	0	0	0	0	0	0	0	0	0	0	0	0	0	0	0	0	0	0	0	0	0	0	0	0
5	S	S	T	0	0	0	0	0	0	0	0	0	0	0	0	0	0	0	0	0	0	0	0	0	0	0	0
6	G	G	T	0	0	0	0	0	0	0	0	0	0	0	0	0	0	0	0	0	0	0	0	0	0	0	0
7	G	G	T	0	0	0	0	0	0	0	0	0	0	0	0	0	0	0	0	0	0	0	0	0	0	0	0
8	S	S	T	0	0	0	0	0	0	0	0	0	0	0	0	0	0	0	0	0	0	0	0	0	0	0	0
9	G	G	T	0	0	0	0	0	0	0	0	0	0	0	0	0	0	0	0	0	0	0	0	0	0	0	0
10	G	G	T	0	0	0	0	0	0	0	0	0	0	0	0	0	0	0	0	0	0	0	0	0	0	0	0
11	G	G	T	0	0	0	0	0	0	0	0	0	0	0	0	0	0	0	0	0	0	0	0	0	0	0	0
12	V	V	T	0	0	0	0	0	0	0	0	0	0	0	0	0	0	0	0	0	0	0	0	0	0	0	0
13	P	P	T	0	0	0	0	0	0	0	0	0	0	0	0	0	0	0	0	0	0	0	0	0	0	0	0
14	E	E	T	0	0	0	0	0	0	0	0	0	0	0	0	0	0	0	0	0	0	0	0	0	0	0	0
15	Q	Q	T	0	0	0	0	0	0	0	0	0	0	0	0	0	0	0	0	0	0	0	0	0	0	0	0
16	E	E	T	0	0	0	0	0	0	0	0	0	0	0	0	0	0	0	0	0	0	0	0	0	0	0	0
17	D	D	T	0	0	0	0	0	0	0	0	0	0	0	0	0	0	0	0	0	0	0	0	0	0	0	0
18	S	S	T	0	0	0	0	0	0	0	0	0	0	0	0	0	0	0	0	0	0	0	0	0	0	0	0
19	V	V	T	0	0	0	0	0	0	0	0	0	0	0	0	0	0	0	0	0	0	0	0	0	0	0	0
20	L	L	T	0	0	0	0	0	0	0	0	0	0	0	0	0	0	0	0	0	0	0	0	0	0	0	0
21	F	F	T	0	0	0	0	0	0	0	0	0	0	0	0	0	0	0	0	0	0	0	0	0	0	0	0
22	R	R	T	0	0	0	0	0	0	0	0	0	0	0	0	0	0	0	0	0	0	0	0	0	0	0	0
23	R	R	T	0	0	0	0	0	0	0	0	0	0	0	0	0	0	0	0	0	0	0	0	0	0	0	0
24	G	G	T	0	0	0	0	0	0	0	0	0	0	0	0	0	0	0	0	0	0	0	0	0	0	0	0
25	T	T	T	0	0	0	0	0	0	0	0	0	0	0	0	0	0	0	0	0	0	0	0	0	0	0	0
26	G	G	T	0	0	0	0	0	0	0	0	0	0	0	0	123.1	10.9	80.1	1.54	0	0	0	0	0	0	0	0
27	Q	Q	T	0	0	0	0	0	0	0	0	0	0	0	0	188.2	15.56	178.5	1.05	0	0	0	0	0	0	0	0
28	S	S	T	0	0	0	0	0	0	0	0	0	0	0	0	98.16	11.23	116.5	0.84	0	0	0	0	0	0	0	0
29	D	D	T	0	0	0	0	0	0	0	0	0	0	0	0	127.9	21.14	140.3	0.91	0	0	0	0	0	0	0	0
30	D	D	T	0	0	0	0	0	0	0	0	0	0	0	0	111.8	16.85	140.3	0.8	0	0	0	0	0	0	0	0
31	S	S	T	0	0	0	0	0	1	0	0	0	0	0	0	70.06	22.22	116.5	0.6	0	0	0	0	0	0	0	0
32	D	D	T	0	6	0	0	0	2	0	0	0	0	0	0	106.3	26.93	140.3	0.76	0	0	0	0	0	0	0	0
33	I	I	T	0	0	0	0	0	0	0	0	0	0	0	0	57.06	17.7	175.1	0.33	0	0	0	0	0	0	0	0
34	W	W	T	0	0	0	0	0	0	0	0	0	0	0	0	159.6	22.19	249.3	0.64	0	0	0	0	0	0	0	0
35	D	D	T	0	24	0	0	0	0	0	0	0	0	0	0	54.34	20.18	140.3	0.39	0	0	0	0	0	0	0	0
36	D	D	T	0	2	0	0	0	1	0	0	0	0	0	0	28.44	13.25	140.3	0.2	0	0	0	0	0	0	0	0
37	T	T	T	0	0	0	0	0	0	0	0	0	0	0	0	56.94	7.86	139.2	0.41	0	0	0	0	0	0	0	0
38	A	A	T	0	0	0	0	0	0	0	0	0	0	0	0	13.31	6.63	107.9	0.12	0	0	0	0	0	0	0	0
39	L	L	T	0	0	0	0	0	0	0	0	0	0	0	0	37.28	5.94	178.6	0.21	0	0	0	0	0	0	0	0
40	I	I	T	0	0	0	0	0	0	0	0	0	0	0	0	93.69	13.86	175.1	0.53	0	0	0	0	0	0	0	0
41	K	K	T	0	15	0	0	0	0	0	0	0	0	0	0	119.3	12.56	200.8	0.59	0	0	0	0	0	0	0	0
42	A	A	T	0	0	0	0	0	0	0	0	0	0	0	0	11.19	6.36	107.9	0.1	0	0	0	0	0	0	0	0
43	Y	Y	T	0	0	0	0	0	0	0	0	0	0	0	0	43.5	11.58	212.7	0.2	0	0	0	0	0	0	0	0
44	D	D	T	0	3	0	0	0	0	0	0	0	0	0	0	101.1	9.16	140.3	0.72	0	0	0	0	0	0	0	0
45	K	K	T	0	0	0	0	0	1	0	0	0	0	0	0	80.56	13.12	200.8	0.4	0	0	0	0	0	0	0	0
46	A	A	T	0	0	0	0	0	0	0	0	0	0	0	0	11.69	5.4	107.9	0.11	0	0	0	0	0	0	0	0
47	V	V	T	0	0	0	0	0	0	0	0	0	0	0	0	58.41	9.22	151.4	0.39	0	0	0	0	0	0	0	0
48	A	A	T	0	0	0	0	0	0	0	0	0	0	0	0	44.75	8.04	107.9	0.41	0	0	0	0	0	0	0	0
49	S	S	T	0	0	0	0	0	0	0	0	0	0	0	0	39.12	12.29	116.5	0.34	0	0	0	0	0	0	0	0
50	F	F	T	0	0	0	0	0	0	0	0	0	0	0	0	107.6	22.71	199.4	0.54	0	0	0	0	0	0	0	0
51	K	K	T	0	0	0	0	0	0	0	0	0	0	0	0	212.1	36.23	200.8	1.06	0	0	0	0	0	0	0	0
52	H	H	T	0	0	0	0	0	0	0	0	0	0	0	0	0	0	0	0	0	0	0	0	0	0	0	0
53	A	A	T	0	0	0	0	0	0	0	0	0	0	0	0	0	0	0	0	0	0	0	0	0	0	0	0
54	L	L	T	0	0	0	0	0	0	0	0	0	0	0	0	0	0	0	0	0	0	0	0	0	0	0	0
55	K	K	T	0	0	0	0	0	0	0	0	0	0	0	0	0	0	0	0	0	0	0	0	0	0	0	0
56	N	N	T	0	0	0	0	0	0	0	0	0	0	0	0	0	0	0	0	0	0	0	0	0	0	0	0
57	G	G	T	0	0	0	0	0	0	0	0	0	0	0	0	0	0	0	0	0	0	0	0	0	0	0	0
58	D	D	T	0	0	0	0	0	0	0	0	0	0	0	0	0	0	0	0	0	0	0	0	0	0	0	0
59	I	I	T	0	0	0	0	0	0	0	0	0	0	0	0	0	0	0	0	0	0	0	0	0	0	0	0
60	C	C	T	0	0	0	0	0	0	0	0	0	0	0	0	0	0	0	0	0	0	0	0	0	0	0	0
61	E	E	T	0	0	0	0	0	0	0	0	0	0	0	0	0	0	0	0	0	0	0	0	0	0	0	0
62	T	T	T	0	0	0	0	0	0	0	0	0	0	0	0	0	0	0	0	0	0	0	0	0	0	0	0
63	S	S	T	0	0	0	0	0	0	0	0	0	0	0	0	0	0	0	0	0	0	0	0	0	0	0	0
64	G	G	T	0	0	0	0	0	0	0	0	0	0	0	0	0	0	0	0	0	0	0	0	0	0	0	0
65	K	K	T	0	0	0	0	0	0	0	0	0	0	0	0	0	0	0	0	0	0	0	0	0	0	0	0
66	P	P	T	0	0	0	0	0	0	0	0	0	0	0	0	0	0	0	0	0	0	0	0	0	0	0	0
67	K	K	T	0	0	0	0	0	0	0	0	0	0	0	0	0	0	0	0	0	0	0	0	0	0	0	0
68	T	T	T	0	0	0	0	0	0	0	0	0	0	0	0	0	0	0	0	0	0	0	0	0	0	0	0
69	T	T	T	0	0	0	0	0	0	0	0	0	0	0	0	0	0	0	0	0	0	0	0	0	0	0	0
70	P	P	T	0	0	0	0	0	0	0	0	0	0	0	0	0	0	0	0	0	0	0	0	0	0	0	0
71	K	K	T	0	0	0	0	0	0	0	0	0	0	0	0	0	0	0	0	0	0	0	0	0	0	0	0
72	R	R	T	0	0	0	0	0	0	0	0	0	0	0	0	0	0	0	0	0	0	0	0	0	0	0	0
73	K	K	T	0	0	0	0	0	0	0	0	0	0	0	0	0	0	0	0	0	0	0	0	0	0	0	0
74	P	P	T	0	0	0	0	0	0	0	0	0	0	0	0	0	0	0	0	0	0	0	0	0	0	0	0
75	A	A	T	0	0	0	0	0	0	0	0	0	0	0	0	0	0	0	0	0	0	0	0	0	0	0	0
76	K	K	T	0	0	0	0	0	0	0	0	0	0	0	0	0	0	0	0	0	0	0	0	0	0	0	0
77	K	K	T	0	0	0	0	0	0	0	0	0	0	0	0	0	0	0	0	0	0	0	0	0	0	0	0
78	N	N	T	0	0	0	0	0	0	0	0	0	0	0	0	0	0	0	0	0	0	0	0	0	0	0	0
79	K	K	T	0	0	0	0	0	0	0	0	0	0	0	0	0	0	0	0	0	0	0	0	0	0	0	0
80	S	S	T	0	0	0	0	0	0	0	0	0	0	0	0	0	0	0	0	0	0	0	0	0	0	0	0
81	Q	Q	T	0	0	0	0	0	0	0	0	0	0	0	0	0	0	0	0	0	0	0	0	0	0	0	0

82	K	K	T	0	0	0	0	0	0	0	0	0	0	0	0	0	0	0	0	0	0	0	0	0	0	0	0
83	K	K	T	0	0	0	0	0	0	0	0	0	0	0	0	0	0	0	0	0	0	0	0	0	0	0	0
84	N	N	T	0	0	0	0	0	0	0	3	0	0	0	0	0	0	0	0	171.5	32.32	143.9	1.19	171.2	52.83	143.9	1.19
85	T	T	T	0	0	0	0	0	0	3	1	0	0	0	0	0	0	0	0	109.2	32	139.2	0.78	116.4	23.19	139.2	0.84
86	A	A	T	0	0	0	0	0	0	0	0	0	0	0	0	0	0	0	0	93.65	16.91	107.9	0.87	88.4	18.05	107.9	0.82
87	A	A	T	0	0	0	0	0	0	0	0	0	0	0	0	0	0	0	0	58.2	20.28	107.9	0.54	41.2	11.96	107.9	0.38
88	S	S	T	0	0	0	0	0	0	1	2	0	0	0	0	0	0	0	0	68.05	23.93	116.5	0.58	66.35	16.42	116.5	0.57
89	L	L	T	0	0	0	0	0	0	0	0	0	0	0	0	0	0	0	0	64.3	19.34	178.6	0.36	145.2	6.77	178.6	0.81
90	Q	Q	T	0	0	0	0	0	0	6	1	204.4	15.6	178.5	1.15	0	0	0	0	95.7	28.46	178.5	0.54	58.75	20.8	178.5	0.33
91	Q	Q	T	0	0	0	0	0	0	0	0	138.6	15.9	178.5	0.78	0	0	0	0	132.4	17.74	178.5	0.74	139.2	7.29	178.5	0.78
92	W	W	T	0	0	0	0	0	0	0	0	66.7	12	249.3	0.27	0	0	0	0	35.65	4.88	249.3	0.14	23.9	5.78	249.3	0.1
93	K	K	T	1	0	18	16	0	0	17	16	125.7	10.77	200.8	0.63	0	0	0	0	127.7	4.07	200.8	0.64	127.0	6.21	200.8	0.63
94	V	V	T	0	0	0	0	0	0	0	0	94.2	9.31	151.4	0.62	0	0	0	0	80.95	2.14	151.4	0.53	85.1	2.75	151.4	0.56
95	G	G	T	0	0	0	0	0	0	0	0	52.2	3.55	80.1	0.65	0	0	0	0	52.85	3.48	80.1	0.66	51.8	1.64	80.1	0.65
96	D	D	T	1	0	20	16	0	0	17	16	57.6	5.5	140.3	0.41	0	0	0	0	49.8	6.75	140.3	0.35	40.35	6.29	140.3	0.29
97	K	K	T	0	0	4	15	0	0	4	18	118.8	11.22	200.8	0.59	0	0	0	0	114.5	17.43	200.8	0.57	92.45	17.14	200.8	0.46
98	C	C	T	0	0	0	0	0	0	0	0	2.8	0.79	134.2	0.02	0	0	0	0	0.9	0.64	134.2	0.01	1.1	0.55	134.2	0.01
99	S	S	T	0	0	0	0	0	0	0	0	12.8	8.44	116.5	0.11	0	0	0	0	0.6	0.88	116.5	0.01	2.1	2.15	116.5	0.02
100	A	A	T	0	0	0	0	0	0	0	0	0.5	0.71	107.9	0.0	0	0	0	0	0.4	0.5	107.9	0.0	0.05	0.22	107.9	0.0
101	I	I	T	0	0	0	0	0	0	0	0	58.4	9.49	175.1	0.33	0	0	0	0	51.5	2.21	175.1	0.29	62.15	7.26	175.1	0.35
102	W	W	T	0	0	0	0	0	0	0	0	51.8	12.88	249.3	0.21	0	0	0	0	17.5	5.91	249.3	0.07	33.1	2.29	249.3	0.13
103	S	S	T	0	0	0	0	0	0	18	12	106	14.01	116.5	0.91	0	0	0	0	78	4.32	116.5	0.67	83.35	2.46	116.5	0.72
104	E	E	T	0	0	0	0	0	0	0	0	137.5	11.05	172.2	0.8	0	0	0	0	142.2	4.96	172.2	0.83	147.0	2.24	172.2	0.85
105	D	D	T	0	0	0	0	0	0	10	0	124.2	15.96	140.3	0.88	0	0	0	0	76.75	6.73	140.3	0.55	96.85	2.83	140.3	0.69
106	G	G	T	0	0	0	0	0	0	0	0	53.3	2.67	80.1	0.67	0	0	0	0	44.95	1	80.1	0.56	41.15	0.93	80.1	0.51
107	C	C	T	0	0	0	0	0	0	0	0	57.1	8.49	134.2	0.43	0	0	0	0	59.6	3.35	134.2	0.44	54.75	1.37	134.2	0.41
108	I	I	T	0	0	0	0	0	0	0	0	105.7	8.34	175.1	0.6	0	0	0	0	90.5	4.32	175.1	0.52	73.5	6.19	175.1	0.42
109	Y	Y	T	0	0	0	0	0	0	10	0	61.1	8.43	212.7	0.29	0	0	0	0	36.9	3.61	212.7	0.17	50.5	2.33	212.7	0.24
110	P	P	T	0	0	0	0	0	0	0	0	63	6.31	136.1	0.46	0	0	0	0	27.1	10.62	136.1	0.2	26.8	7.43	136.1	0.2
111	A	A	T	0	0	0	0	0	0	0	0	0.3	0.48	107.9	0.0	0	0	0	0	0.9	0.31	107.9	0.01	0.85	0.37	107.9	0.01
112	T	T	T	0	0	0	0	0	0	2	1	41.9	4.58	139.2	0.3	0	0	0	0	38	3.29	139.2	0.27	38.2	4.14	139.2	0.27
113	I	I	T	0	0	0	0	0	0	0	0	1.4	0.84	175.1	0.01	0	0	0	0	3.75	0.85	175.1	0.02	4	0.65	175.1	0.02
114	A	A	T	0	0	0	0	0	0	0	0	41.8	7.96	107.9	0.39	0	0	0	0	46.2	4.5	107.9	0.43	43.2	2.65	107.9	0.4
115	S	S	T	0	0	0	0	0	0	0	0	56.5	9.86	116.5	0.48	0	0	0	0	63	8.11	116.5	0.54	66.75	4.55	116.5	0.57
116	I	I	T	0	0	0	0	0	0	0	0	26.6	4.03	175.1	0.15	0	0	0	0	23.8	1.58	175.1	0.14	25.45	1.28	175.1	0.15
117	D	D	T	1	0	8	5	0	0	5	3	76.8	15.98	140.3	0.55	0	0	0	0	58.8	9.83	140.3	0.42	58.2	4.93	140.3	0.41
118	F	F	T	0	0	0	0	0	0	0	0	156.1	18.72	199.4	0.78	0	0	0	0	138.5	8	199.4	0.69	120.5	8.49	199.4	0.6
119	K	K	T	1	0	7	0	0	0	4	0	178.3	14.6	200.8	0.89	0	0	0	0	176.0	12.37	200.8	0.88	196.1	4.8	200.8	0.98
120	R	R	T	0	0	1	24	0	0	1	19	200.9	18.81	238.7	0.84	0	0	0	0	185.3	8.97	238.7	0.78	150.4	11.7	238.7	0.63
121	E	E	T	0	0	0	0	0	0	0	11	121.6	6.8	172.2	0.71	0	0	0	0	117.5	14.87	172.2	0.68	98.8	10.06	172.2	0.57
122	T	T	T	0	0	0	0	0	0	6	17	22.7	4.69	139.2	0.16	0	0	0	0	12.3	1.3	139.2	0.09	13.1	1.97	139.2	0.09
123	C	C	T	0	0	0	0	0	0	0	0	1.3	1.25	134.2	0.01	0	0	0	0	1	0.32	134.2	0.01	1	0.56	134.2	0.01
124	V	V	T	0	0	0	0	0	0	0	0	32.6	5.82	151.4	0.22	0	0	0	0	21.45	6.52	151.4	0.14	28.45	5.1	151.4	0.19
125	V	V	T	0	0	0	0	0	0	0	0	0.1	0.32	151.4	0.0	0	0	0	0	0	0	151.4	0.0	0	0	151.4	0.0
126	V	V	T	0	0	0	0	0	0	0	0	39.6	5.15	151.4	0.26	0	0	0	0	53.05	1.39	151.4	0.35	49.8	1.96	151.4	0.33
127	Y	Y	T	0	0	0	0	0	0	20	33	2.7	1.77	212.7	0.01	0	0	0	0	0.25	0.44	212.7	0.0	0.8	0.41	212.7	0.0
128	T	T	T	0	0	0	0	0	0	2	0	60.6	5.93	139.2	0.44	0	0	0	0	60.4	8.26	139.2	0.43	53	10.21	139.2	0.38
129	G	G	T	0	0	0	0	0	0	0	0	80	5.46	80.1	1	0	0	0	0	81.3	1.81	80.1	1.01	82.05	1.19	80.1	1.02
130	Y	Y	T	0	0	0	0	0	0	0	0	119.2	11.16	212.7	0.56	0	0	0	0	111.1	4.18	212.7	0.52	105.2	3.34	212.7	0.49
131	G	G	T	0	0	0	0	0	0	0	0	63.2	13.05	80.1	0.79	0	0	0	0	56.3	1.56	80.1	0.7	55.4	1.98	80.1	0.69
132	N	N	T	0	0	0	0	0	0	0	20	73.4	12.16	143.9	0.51	0	0	0	0	77.1	3.48	143.9	0.54	31.6	1.82	143.9	0.22
133	R	R	T	0	0	0	0	0	0	0	0	167.3	12.84	238.7	0.7	0	0	0	0	180.2	3.85	238.7	0.75	186.8	2.98	238.7	0.78
134	E	E	T	0	0	0	0	0	0	38	31	55.4	20.08	172.2	0.32	0	0	0	0	36.35	3.05	172.2	0.21	34.25	5.62	172.2	0.2
135	E	E	T	0	0	0	19	0	0	6	33	119.5	9.48	172.2	0.69	0	0	0	0	112.7	6.31	172.2	0.65	82.45	5.17	172.2	0.48
136	Q	Q	T	0	0	0	0	0	0	0	14	30.1	7.99	178.5	0.17	0	0	0	0	13.55	1.43	178.5	0.08	12.4	4.65	178.5	0.07
137	N	N	T	0	0	0	0	0	0	0	0	69.6	9.01	143.9	0.48	0	0	0	0	74.8	4.82	143.9	0.52	68.6	9.18	143.9	0.48
138	L	L	T	0	0	0	0	0	0	0	0	27	7.5	178.6	0.15	0	0	0	0	8.6	3.63	178.6	0.05	9.05	3.5	178.6	0.05
139	S	S	T	0	0	0	0	0	0	4	13	105.4	6.24	116.5	0.9	0	0	0	0	69.95	12.88	116.5	0.6	26.95	19.25	116.5	0.23
140	D	D	T	0	0	0	0	0	0	0	2	81.3	9.86	140.3	0.58	0	0	0	0	75.3	3.84	140.3	0.54	77.4	15.89	140.3	0.55
141	L	L	T	0	0	0	0	0	0	0	0	17.4	2.5	178.6	0.1	0	0	0	0	2.05	0.83	178.6	0.01	3.1	3.4	178.6	0.02
142	L	L	T	0	0	0	0	0	0	0	0	63.4	6.47	178.6	0.35	0	0	0	0	54.1	3.01	178.6	0.3	65.65	10.97	178.6	0.37
143	S	S	T	0	0	0	0	0	0	0	1	81.9	7.65	116.5	0.7	0	0	0	0	75.15	13.8	116.5	0.65	87.1	8.58	116.5	0.75
144	P	P	T	0	0	0	0	0	0	0	0	75.3	6.15	136.1	0.55	0	0	0	0	61.65	9.64	136.1	0.45	52.4	6.75	136.1	0.38
145	I	I	T	0	0	0	0	0	0	0	0	196.8	17.36	175.1	1.12	0	0	0	0	71	9.08	175.1	0.41	87.8	13.24	175.1	0.5
146	C	C	T	0	0	0	0	0	0	0	0	0	0	0	0	0	0	0	0	122.3	10.69	134.2	0.91	125.1	8.7	134.2	0.93
147	E	E	T	0	0	2	15	0	0	4	17	0	0	0	0	0	0	0	0	208.4	37.23	172.2	1.21	173.6	22.31	172.2	1.01
148	V	V	T	0	0	0	0	0	0	0	0	0	0	0	0	0	0	0	0	0	0	0	0	0	0	0	0
149	A	A	T	0	0	0	0	0	0	0	0	0	0	0	0	0	0	0	0	0	0	0	0	0	0	0	0
150	N	N	T	0	0	0	0	0	0	0	0	0	0	0	0	0	0	0	0	0	0	0	0	0	0	0	0
151	N	N	T	0	0	0	0	0	0	0	0	0	0	0	0	0	0	0	0	0	0	0	0	0	0	0	0
152	I	I	T	0	0	0	0	0	0	0	0	0	0	0	0	0	0	0	0	0	0	0	0	0	0	0	0
153	E	E	T	0	0	0	0	0	0	0	0	0	0	0	0	0	0	0	0	0	0	0	0	0	0	0	0
154	Q	Q	T	0	0	0	0	0	0	0	0	0	0	0	0	0	0	0	0	0	0	0	0	0	0	0	0
155	N	N	T	0	0	0	0	0	0	0	0	0	0	0	0	0	0	0	0	0	0	0	0	0	0	0	0
156	A	A	T	0	0	0	0	0	0	0	0	0	0	0	0	0	0	0	0	0	0	0	0	0	0	0	0
157	Q	Q	T	0	0	0	0	0	0	0	0	0	0	0	0	0	0	0	0	0	0	0	0	0	0	0	0
158	E	E	T	0	0	0	0	0	0	0	0	0	0	0	0	0	0	0	0	0	0	0	0	0	0	0	0
159	N	N	T	0	0	0	0	0	0	0	0	0	0	0	0	0	0	0	0	0	0	0	0	0	0	0	0
160	E	E	T	0	0	0	0	0	0	0	0	0	0	0	0	0	0	0	0	0	0	0	0	0	0	0	0
161	N	N	T	0	0	0	0	0	0	0	0	0	0	0	0	0	0	0	0	0	0	0	0	0	0	0	0
162	E	E	T	0	0	0	0	0	0	0	0	0	0	0	0	0	0	0	0	0	0	0	0	0	0	0	0
163	S	S	T	0	0	0	0	0	0	0	0	0	0	0	0	0	0	0	0	0	0	0	0	0	0	0	0
164	Q	Q	T	0	0	0	0	0	0	0	0	0	0	0	0	0	0	0	0	0	0	0	0	0	0	0	0

165	V	V	T	0	0	0	0	0	0	0	0	0	0	0	0	0	0	0	0	0	0	0	0	0	0	0	0
166	S	S	T	0	0	0	0	0	0	0	0	0	0	0	0	0	0	0	0	0	0	0	0	0	0	0	0
167	T	T	T	0	0	0	0	0	0	0	0	0	0	0	0	0	0	0	0	0	0	0	0	0	0	0	0
168	D	D	T	0	0	0	0	0	0	0	0	0	0	0	0	0	0	0	0	0	0	0	0	0	0	0	0
169	E	E	T	0	0	0	0	0	0	0	0	0	0	0	0	0	0	0	0	0	0	0	0	0	0	0	0
170	S	S	T	0	0	0	0	0	0	0	0	0	0	0	0	0	0	0	0	0	0	0	0	0	0	0	0
171	E	E	T	0	0	0	0	0	0	0	0	0	0	0	0	0	0	0	0	0	0	0	0	0	0	0	0
172	N	N	T	0	0	0	0	0	0	0	0	0	0	0	0	0	0	0	0	0	0	0	0	0	0	0	0
173	S	S	T	0	0	0	0	0	0	0	0	0	0	0	0	0	0	0	0	0	0	0	0	0	0	0	0
174	R	R	T	0	0	0	0	0	0	0	0	0	0	0	0	0	0	0	0	0	0	0	0	0	0	0	0
175	S	S	T	0	0	0	0	0	0	0	0	0	0	0	0	0	0	0	0	0	0	0	0	0	0	0	0
176	P	P	T	0	0	0	0	0	0	0	0	0	0	0	0	0	0	0	0	0	0	0	0	0	0	0	0
177	G	G	T	0	0	0	0	0	0	0	0	0	0	0	0	0	0	0	0	0	0	0	0	0	0	0	0
178	N	N	T	0	0	0	0	0	0	0	0	0	0	0	0	0	0	0	0	0	0	0	0	0	0	0	0
179	K	K	T	0	0	0	0	0	0	0	0	0	0	0	0	0	0	0	0	0	0	0	0	0	0	0	0
180	S	S	T	0	0	0	0	0	0	0	0	0	0	0	0	0	0	0	0	0	0	0	0	0	0	0	0
181	D	D	T	0	0	0	0	0	0	0	0	0	0	0	0	0	0	0	0	0	0	0	0	0	0	0	0
182	N	N	T	0	0	0	0	0	0	0	0	0	0	0	0	0	0	0	0	0	0	0	0	0	0	0	0
183	I	I	T	0	0	0	0	0	0	0	0	0	0	0	0	0	0	0	0	0	0	0	0	0	0	0	0
184	K	K	T	0	0	0	0	0	0	0	0	0	0	0	0	0	0	0	0	0	0	0	0	0	0	0	0
185	P	P	T	0	0	0	0	0	0	0	0	0	0	0	0	0	0	0	0	0	0	0	0	0	0	0	0
186	K	K	T	0	0	0	0	0	0	0	0	0	0	0	0	0	0	0	0	0	0	0	0	0	0	0	0
187	S	S	T	0	0	0	0	0	0	0	0	0	0	0	0	0	0	0	0	0	0	0	0	0	0	0	0
188	A	A	T	0	0	0	0	0	0	0	0	0	0	0	0	0	0	0	0	0	0	0	0	0	0	0	0
189	P	P	T	0	0	0	0	0	0	0	0	0	0	0	0	0	0	0	0	0	0	0	0	0	0	0	0
190	W	W	T	0	0	0	0	0	0	0	0	0	0	0	0	0	0	0	0	0	0	0	0	0	0	0	0
191	N	N	T	0	0	0	0	0	0	0	0	0	0	0	0	0	0	0	0	0	0	0	0	0	0	0	0
192	S	S	T	0	0	0	0	0	0	0	0	0	0	0	0	0	0	0	0	0	0	0	0	0	0	0	0
193	F	F	T	0	0	0	0	0	0	0	0	0	0	0	0	0	0	0	0	0	0	0	0	0	0	0	0
194	L	L	T	0	0	0	0	0	0	0	0	0	0	0	0	0	0	0	0	0	0	0	0	0	0	0	0
195	P	P	T	0	0	0	0	0	0	0	0	0	0	0	0	0	0	0	0	0	0	0	0	0	0	0	0
196	P	P	T	0	0	0	0	0	0	0	0	0	0	0	0	0	0	0	0	0	0	0	0	0	0	0	0
197	P	P	T	0	0	0	0	0	0	0	0	0	0	0	0	0	0	0	0	0	0	0	0	0	0	0	0
198	P	P	T	0	0	0	0	0	0	0	0	0	0	0	0	0	0	0	0	0	0	0	0	0	0	0	0
199	P	P	T	0	0	0	0	0	0	0	0	0	0	0	0	0	0	0	0	0	0	0	0	0	0	0	0
200	M	M	T	0	0	0	0	0	0	0	0	0	0	0	0	0	0	0	0	0	0	0	0	0	0	0	0
201	P	P	T	0	0	0	0	0	0	0	0	0	0	0	0	0	0	0	0	0	0	0	0	0	0	0	0
202	G	G	T	0	0	0	0	0	0	0	0	0	0	0	0	0	0	0	0	0	0	0	0	0	0	0	0
203	P	P	T	0	0	0	0	0	0	0	0	0	0	0	0	0	0	0	0	0	0	0	0	0	0	0	0
204	R	R	T	0	0	0	0	0	0	0	0	0	0	0	0	0	0	0	0	0	0	0	0	0	0	0	0
205	L	L	T	0	0	0	0	0	0	0	0	0	0	0	0	0	0	0	0	0	0	0	0	0	0	0	0
206	G	G	T	0	0	0	0	0	0	0	0	0	0	0	0	0	0	0	0	0	0	0	0	0	0	0	0
207	P	P	T	0	0	0	0	0	0	0	0	0	0	0	0	0	0	0	0	0	0	0	0	0	0	0	0
208	G	G	T	0	0	0	0	0	0	0	0	0	0	0	0	0	0	0	0	0	0	0	0	0	0	0	0
209	K	K	T	0	0	0	0	0	0	0	0	0	0	0	0	0	0	0	0	0	0	0	0	0	0	0	0
210	P	P	T	0	0	0	0	0	0	0	0	0	0	0	0	0	0	0	0	0	0	0	0	0	0	0	0
211	G	G	T	0	0	0	0	0	0	0	0	0	0	0	0	0	0	0	0	0	0	0	0	0	0	0	0
212	L	L	T	0	0	0	0	0	0	0	0	0	0	0	0	0	0	0	0	0	0	0	0	0	0	0	0
213	K	K	T	0	0	0	0	0	0	0	0	0	0	0	0	0	0	0	0	0	0	0	0	0	0	0	0
214	F	F	T	0	0	0	0	0	0	0	0	0	0	0	0	0	0	0	0	0	0	0	0	0	0	0	0
215	N	N	T	0	0	0	0	0	0	0	0	0	0	0	0	0	0	0	0	0	0	0	0	0	0	0	0
216	G	G	T	0	0	0	0	0	0	0	0	0	0	0	0	0	0	0	0	0	0	0	0	0	0	0	0
217	P	P	T	0	0	0	0	0	0	0	0	0	0	0	0	0	0	0	0	0	0	0	0	0	0	0	0
218	P	P	T	0	0	0	0	0	0	0	0	0	0	0	0	0	0	0	0	0	0	0	0	0	0	0	0
219	P	P	T	0	0	0	0	0	0	0	0	0	0	0	0	0	0	0	0	0	0	0	0	0	0	0	0
220	P	P	T	0	0	0	0	0	0	0	0	0	0	0	0	0	0	0	0	0	0	0	0	0	0	0	0
221	P	P	T	0	0	0	0	0	0	0	0	0	0	0	0	0	0	0	0	0	0	0	0	0	0	0	0
222	P	P	T	0	0	0	0	0	0	0	0	0	0	0	0	0	0	0	0	0	0	0	0	0	0	0	0
223	P	P	T	0	0	0	0	0	0	0	0	0	0	0	0	0	0	0	0	0	0	0	0	0	0	0	0
224	P	P	T	0	0	0	0	0	0	0	0	0	0	0	0	0	0	0	0	0	0	0	0	0	0	0	0
225	P	P	T	0	0	0	0	0	0	0	0	0	0	0	0	0	0	0	0	0	0	0	0	0	0	0	0
226	P	P	T	0	0	0	0	0	0	0	0	0	0	0	0	0	0	0	0	0	0	0	0	0	0	0	0
227	H	H	T	0	0	0	0	0	0	0	0	0	0	0	0	0	0	0	0	0	0	0	0	0	0	0	0
228	L	L	T	0	0	0	0	0	0	0	0	0	0	0	0	0	0	0	0	0	0	0	0	0	0	0	0
229	L	L	T	0	0	0	0	0	0	0	0	0	0	0	0	0	0	0	0	0	0	0	0	0	0	0	0
230	S	S	T	0	0	0	0	0	0	0	0	0	0	0	0	0	0	0	0	0	0	0	0	0	0	0	0
231	C	C	T	0	0	0	0	0	0	0	0	0	0	0	0	0	0	0	0	0	0	0	0	0	0	0	0
232	W	W	T	0	0	0	0	0	0	0	0	0	0	0	0	0	0	0	0	0	0	0	0	0	0	0	0
233	L	L	T	0	0	0	0	0	0	0	0	0	0	0	0	0	0	0	0	0	0	0	0	0	0	0	0
234	P	P	T	0	0	0	0	0	0	0	0	0	0	0	0	0	0	0	0	0	0	0	0	0	0	0	0
235	P	P	T	0	0	0	0	0	0	0	0	0	0	0	0	0	0	0	0	0	0	0	0	0	0	0	0
236	F	F	T	0	0	0	0	0	0	0	0	0	0	0	0	0	0	0	0	0	0	0	0	0	0	0	0
237	P	P	T	0	0	0	0	0	0	0	0	0	0	0	0	0	0	0	0	0	0	0	0	0	0	0	0
238	S	S	T	0	0	0	0	0	0	0	0	0	0	0	0	0	0	0	0	0	0	0	0	0	0	0	0
239	G	G	T	0	0	0	0	0	0	0	0	0	0	0	0	0	0	0	0	0	0	0	0	0	0	0	0
240	P	P	T	0	0	0	0	0	0	0	0	0	0	0	0	0	0	0	0	0	0	0	0	0	0	0	0
241	P	P	T	0	0	0	0	0	0	0	0	0	0	0	0	0	0	0	0	0	0	0	0	0	0	0	0
242	I	I	T	0	0	0	0	0	0	0	0	0	0	0	0	0	0	0	0	0	0	0	0	0	0	0	0
243	I	I	T	0	0	0	0	0	0	0	0	0	0	0	0	0	0	0	0	0	0	0	0	0	0	0	0
244	P	P	T	0	0	0	0	0	0	0	0	0	0	0	0	0	0	0	0	0	0	0	0	0	0	0	0
245	P	P	T	0	0	0	0	0	0	0	0	0	0	0	0	0	0	0	0	0	0	0	0	0	0	0	0
246	P	P	T	0	0	0	0	0	0	0	0	0	0	0	0	0	0	0	0	0	0	0	0	0	0	0	0
247	P	P	T	0	0	0	0	0	0	0	0	0	0	0	0	0	0	0	0	0	0	0	0	0	0	0	0

248	P	P	T	0	0	0	0	0	0	0	0	0	0	0	0	0	0	0	0	0	0	0	0	0	0	0	0
249	I	I	T	0	0	0	0	0	0	0	0	0	0	0	0	0	0	0	0	0	0	0	0	0	0	0	0
250	C	C	T	0	0	0	0	0	0	0	0	0	0	0	0	0	0	0	0	0	0	0	0	0	0	0	0
251	P	P	T	0	0	0	0	0	0	0	0	0	0	0	0	0	0	0	0	0	0	0	0	0	0	0	0
252	D	D	T	0	0	0	0	0	0	0	0	0	0	0	0	0	0	0	0	0	0	0	0	0	0	0	0
253	S	S	T	0	0	0	0	0	0	0	0	0	0	0	0	0	0	0	0	0	0	0	0	0	0	0	0
254	L	L	T	0	0	0	0	0	0	0	0	0	0	0	0	0	0	0	0	0	0	0	0	0	0	0	0
255	D	D	T	0	0	0	0	0	0	0	0	0	0	0	0	0	0	0	0	0	0	0	0	0	0	0	0
256	D	D	T	0	0	0	0	0	0	0	0	0	0	0	0	0	0	0	0	0	0	0	0	0	0	0	0
257	A	A	T	0	0	0	0	0	0	0	0	0	0	0	0	0	0	0	0	0	0	0	0	0	0	0	0
258	D	D	T	0	0	0	0	0	0	0	0	0	0	0	0	0	0	0	0	0	0	0	0	0	0	0	0
259	A	A	T	0	0	0	0	0	0	0	0	0	0	0	0	0	0	0	0	0	0	0	0	0	0	0	0
260	L	L	T	0	0	0	0	0	0	0	0	0	0	0	0	0	0	0	0	0	0	0	0	0	0	0	0
261	G	G	T	0	0	0	0	0	0	0	0	0	0	0	0	0	0	0	0	0	0	0	0	0	0	0	0
262	S	S	T	0	0	0	0	0	0	0	0	0	0	0	0	0	0	0	0	0	0	0	0	0	0	0	0
263	M	M	T	0	0	0	0	0	0	0	0	0	0	0	0	0	0	0	0	0	0	0	0	0	0	0	0
264	L	L	T	0	0	0	0	0	0	0	0	0	0	0	0	0	0	0	0	0	0	0	0	0	0	0	0
265	I	I	T	0	0	0	0	0	0	0	0	0	0	0	0	0	0	0	0	0	0	0	0	0	0	0	0
266	S	S	T	0	0	0	0	0	0	0	0	0	0	0	0	0	0	0	0	0	0	0	0	0	0	0	0
267	W	W	T	0	0	0	0	0	0	0	0	0	0	0	0	0	0	0	0	0	0	0	0	0	0	0	0
268	Y	Y	T	0	0	0	0	0	0	0	0	0	0	0	0	0	0	0	0	0	0	0	0	0	0	0	0
269	M	M	T	0	0	0	0	0	0	0	0	0	0	0	0	0	0	0	0	0	0	0	0	0	0	0	0
270	S	S	T	0	0	0	0	0	0	0	0	0	0	0	0	0	0	0	0	0	0	0	0	0	0	0	0
271	G	G	T	0	0	0	0	0	0	0	0	0	0	0	0	0	0	0	0	0	0	0	0	0	0	0	0
272	Y	Y	T	0	0	0	0	0	0	0	0	0	0	0	0	0	0	0	0	0	0	0	0	0	0	0	0
273	H	H	T	0	0	0	0	0	0	0	0	0	0	0	0	0	0	0	0	0	0	0	0	0	0	0	0
274	T	T	T	0	0	0	0	0	0	0	0	0	0	0	0	0	0	0	0	0	0	0	0	0	0	0	0
275	G	G	T	0	0	0	0	0	0	0	0	0	0	0	0	0	0	0	0	0	0	0	0	0	0	0	0
276	Y	Y	T	0	0	0	0	0	0	0	0	0	0	0	0	0	0	0	0	0	0	0	0	0	0	0	0
277	Y	Y	T	0	0	0	0	0	0	0	0	0	0	0	0	0	0	0	0	0	0	0	0	0	0	0	0
278	M	M	T	0	0	0	0	0	0	0	0	0	0	0	0	0	0	0	0	0	0	0	0	0	0	0	0
279	G	E	F	0	0	0	0	0	0	0	0	0	0	0	0	0	0	0	0	0	0	0	0	0	0	0	0
280	F	M	F	0	0	0	0	0	0	0	0	0	0	0	0	0	0	0	0	0	0	0	0	0	0	0	0
281	R	L	F	0	0	0	0	0	0	0	0	0	0	0	0	0	0	0	0	0	0	0	0	0	0	0	0
282	Q	A	F	0	0	0	0	0	0	0	0	0	0	0	0	0	0	0	0	0	0	0	0	0	0	0	0
283	N	NA	F	0	0	0	0	0	0	0	0	0	0	0	0	0	0	0	0	0	0	0	0	0	0	0	0
284	Q	NA	F	0	0	0	0	0	0	0	0	0	0	0	0	0	0	0	0	0	0	0	0	0	0	0	0
285	K	NA	F	0	0	0	0	0	0	0	0	0	0	0	0	0	0	0	0	0	0	0	0	0	0	0	0
286	E	NA	F	0	0	0	0	0	0	0	0	0	0	0	0	0	0	0	0	0	0	0	0	0	0	0	0
287	G	NA	F	0	0	0	0	0	0	0	0	0	0	0	0	0	0	0	0	0	0	0	0	0	0	0	0
288	R	NA	F	0	0	0	0	0	0	0	0	0	0	0	0	0	0	0	0	0	0	0	0	0	0	0	0
289	C	NA	F	0	0	0	0	0	0	0	0	0	0	0	0	0	0	0	0	0	0	0	0	0	0	0	0
290	S	NA	F	0	0	0	0	0	0	0	0	0	0	0	0	0	0	0	0	0	0	0	0	0	0	0	0
291	H	NA	F	0	0	0	0	0	0	0	0	0	0	0	0	0	0	0	0	0	0	0	0	0	0	0	0
292	S	NA	F	0	0	0	0	0	0	0	0	0	0	0	0	0	0	0	0	0	0	0	0	0	0	0	0
293	L	NA	F	0	0	0	0	0	0	0	0	0	0	0	0	0	0	0	0	0	0	0	0	0	0	0	0
294	N	NA	F	0	0	0	0	0	0	0	0	0	0	0	0	0	0	0	0	0	0	0	0	0	0	0	0

Table 1 Computational analysis of salt bridge, hydrogen bond and solvent accessible surface area (SASA) for four SMN-related structures determined by solution-state nuclear magnetic resonance (NMR) spectroscopy. In this table, the first column represents the amino acid residue number of SMN1 and SMN2, the second and the third columns represent the amino acid sequences (in one letter code) of SMN1 and SMN2, respectively. In the third column for SMN2, NA represents the absence of amino acid residue for SMN2. In the fourth column, A represents alignment of SMN1 and SMN2 amino acid sequences, T and F represent identical and non-identical alignments for the two sequences, respectively. The next four columns represent for each amino acid residue the number of salt bridges computationally identified in the four NMR emsembles, with numbers 1, 2, 3 and 4 corresponding to the PDB IDs 1g5v, 2leh, 4a4e and 4a4g, respectively. The next four columns represent for each amino acid residue the number of hydrogen bonds computationally identified in the four NMR emsembles, with numbers 1, 2, 3 and 4 corresponding to the PDB IDs 1g5v, 2leh, 4a4e and 4a4g, respectively. In the salt bridge analysis for titrateable residues, 4.0 Å was used as the distance criterion for the two (positively and negatively) charged groups. The hydrogen bond analysis employed a geometric criteria of a cutoff value of the angle formed by acceptor (A), donor (D) and hydrogen (H) $(\angle ADH)$ of 30° and a cutoff of donor-acceptor distance at 3.0 Å, i.e., a hydrogen bond is only considered to be formed if $\angle ADH$ is not larger than 30° and the donor-acceptor distance is not larger than 3.0 Å. In the SASA analysis, $Mean$, Std, $Norm$ and $Ratio$ represent the average SASA value, one standard deviation from the average SASA value, the normal standard SASA value used by the NACCESS program [1], and the ratio of $Mean$ devided by $Normal$. In the computational analysis, an amino acid residue is considered as potentially important only if its side chain is involved in a salt bridge or a hydrogen bond, or its SASA is smaller than 30% of its standard SASA value.

References

1 Simon J Hubbard and Janet M Thornton. Naccess. *Computer Program, Department of Biochemistry and Molecular Biology, University College London*, 2, 1993.
