# Supplementary material for: How do SMA-linked mutations of SMN1 lead to structural/functional deficiency of the SMA protein?
Source: PLoS One. 2017 Jun 1;12(6):e0178519. doi: 10.1371/journal.pone.0178519 (PMC5453535; doi:10.1371/journal.pone.0178519)
Supplement: S2 File — This supplementary file (S2_File.pdf) presents four supporting figures for the visualization of the salt bridge and the hydrogen bond analysis, a table of computationally identified SMN residues aligned against experimentally identified SMA-linked SMN mutations, and a set of tables describing the chemical shift assignment analysis for the SMN-related SMN structures. (PDF) [file pone.0178519.s002.pdf]

1 Supplementary materials

1.1 Results

1.1.1 Salt bridge analysis of the SMN-related structures

M A M S S G G S G G G V P E Q E D S V L F R R G T G Q S D
D S **D** I W **D** **D** T A L I **K** A Y **D** K A V A S F K H A L K
N G D I C E T S G K P K T T P K R K P A K K N K S Q K K N
T A A S L Q Q W **K** V G **D** **K** C S A I W S E D G C I Y P A
T I A S I **D** F **K** R E T C V V V Y T G Y G N R E **E** Q N L S
D L L S P I C **E** V A N N I E Q N A Q E N E N E S Q V S T D
E S E N S R S P G N K S D N I K P K S A P W N S F L P P P
P P M P G P R L G P G K P G L K F N G P P P P P P P P P P
H L L S C W L P P F P S G P P I I P P P P P I C P D S L D D
A D A L G S M L I S W Y M S G Y H T G Y Y M G F R Q N Q
K E G R C S H S L N

Figure 1.1 The amino acid sequence of FL-SMN with computationally identified SB-involved residues highlighted with white texts and black backgrounds.

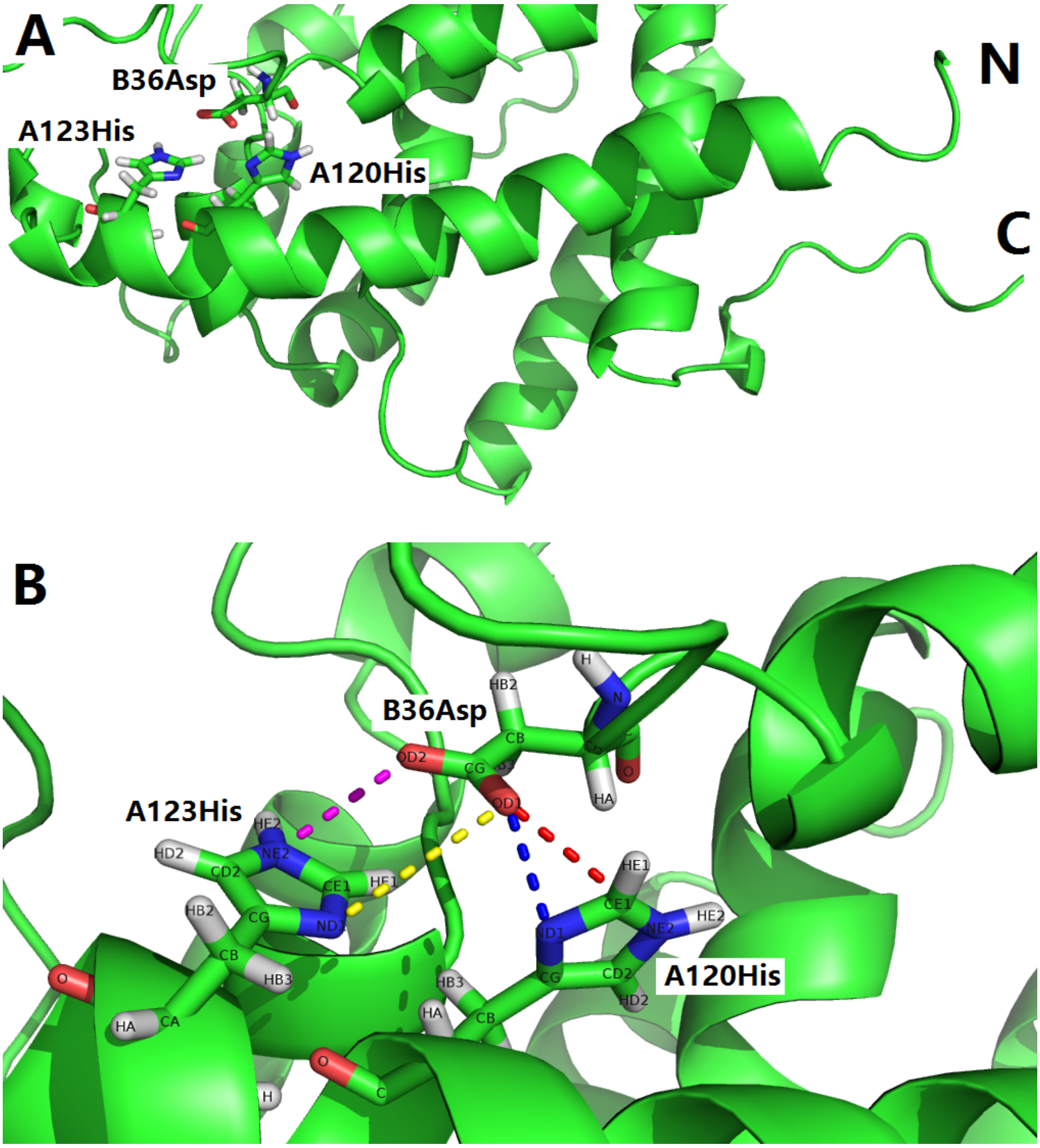

Figure 1.2 Two salt bridge pairs formed between A123His and B36Asp and B36Asp and A120His in the 15th structure model of the NMR ensemble (PDB ID: 2leh) of Solution structure of the core SMN-Gemin2 complex [1]. In this figure, oxygen, nitrogen, carbon and hydrogen atoms are colored red, blue, green and gray, respectively.

1.1.2 Hydrogen bond analysis of the SMN-related structures

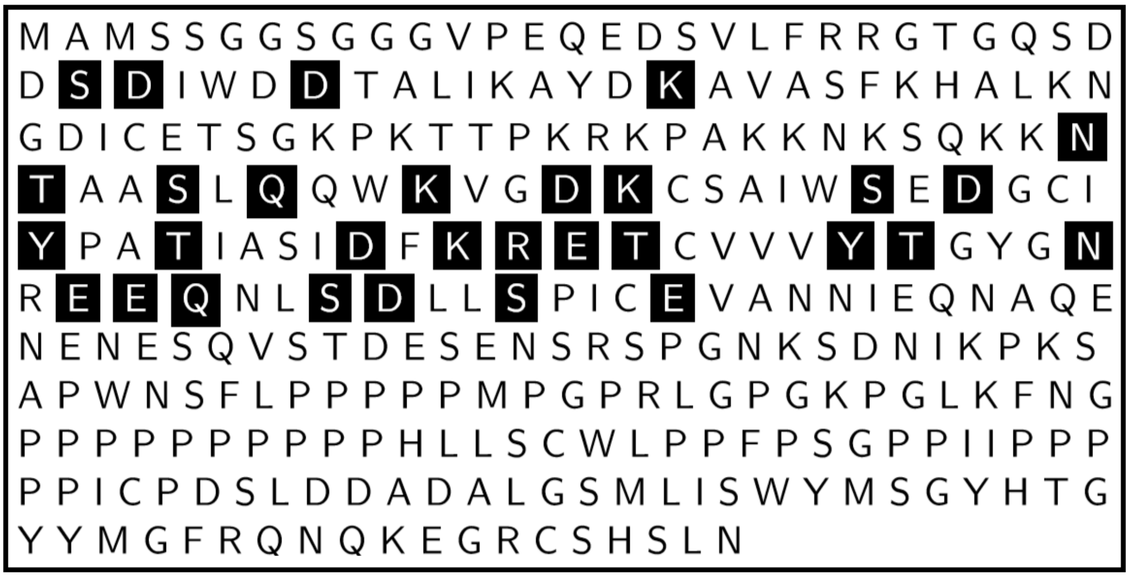

Figure 1.3 The amino acid sequence of FL-SMN with computationally identified HB-involved residues highlighted with white texts and black backgrounds.

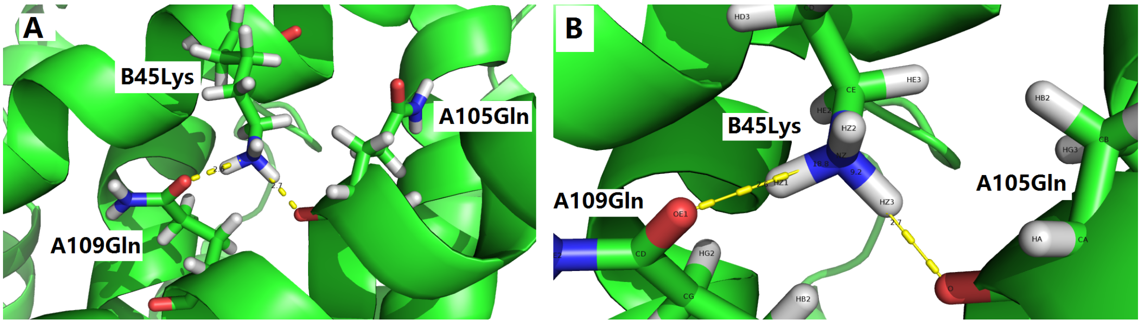

Figure 1.4 Two hydrogen bonds formed between B45Lys and A109Gln and B45Lys and A105Gln in the 8th model in the NMR ensemble (PDB ID: 2leh) Solution structure of the core SMN-Gemin2 complex [1]. In this figure, oxygen, nitrogen, carbon and hydrogen atoms are colored red, blue, green and gray, respectively.

1.1.3 Summary of SMA-linked point mutations of *SMN1*

NMR	X-ray	Literature	Mutation(s)	Reference
		Ala2	Ala2Gly (A2G)	[2]
		Gln15	Gln15X (nonsense mutation Q15X)	[3]
		Asp30	Asp30Asn (D30N)	[4]
Ser31				
Asp32				
Asp35				
Asp36				
Ala38	Ala38			
Leu39	Leu39			
Lys41				
Ala42	Ala42			
Tyr43	Tyr43			
Asp44	Asp44	Asp44	Asp44Val (D44V)	[4]
Lys45				
Ala46	Ala46			
	Val47			
	Ser49			
	Phe50			
Asn84				
Thr85				
Ser88				
Gln90				
Trp92	Trp92	Trp92	Trp92Ser (W92S)	[5]
Lys93	Lys93			
		Val94	Val94Gly (V94G)	[6]
		Gly95	Gly95Arg (G95R)	[4]
Asp96	Asp96			
Lys97				
Cys98	Cys98			
Ser99	Ser99			
Ala100	Ala100			
Ile101				

Trp102	Trp102	Trp102	Trp102X (nonsense mutation W102X)	[7, 8]
Ser103				
Asp105				
Tyr109	Tyr109			
Pro110	Pro110			
Ala111	Ala111	Ala111	Ala111Gly (A111G)	[4, 9]
Thr112	Thr112			
Ile113	Ile113			
Ile116	Ile116	Ile116	Ile116Phe (I116F)	[10]
Asp117				
Lys119				
Arg120				
Glu121				
Thr122	Thr122			
Cys123	Cys123			
Val124	Val124			
Val125	Val125			
Val126	Val126			
Tyr127	Tyr127			
Thr128				
		Tyr130	Tyr130Cys (Y130C)	[7]
Asn132				
Glu134	Glu134	Glu134	Glu134Lys (E134K)	[4]
Glu135				
Gln136	Gln136	Gln136	Gln136Glu (Q136E)	[10]
Leu138	Leu138			
Ser139				
Asp140				
Leu141	Leu141			
	Leu142			
Ser143				
Glu147				
		Gln157	Gln157X (nonsense mutation Q157X)	[11]

		Ala188	Ala188Ser (A188S)	[12]
		Trp190	Trp190X (nonsense mutation W190X)	[13]
		Leu228	Leu228X (nonsense mutation L228X)	[14]
		Pro245	Pro245Leu (P245L)	[15]
		Leu260	Leu260Ser (L260S)	[6]
		Ser262	Ser262Gly, Ser262Ile (S262G,S262I)	[4, 16]
		Met263	Met263Thr (M263T)	[13]
		Ser266	Ser266Pro (S266P)	[7]
		Tyr272	Tyr272Cys (Y272C)	[16–18]
		His273	His273Arg (H273R)	[7]
		Thr274	Thr274Ile (T274I)	[16–18]
		Gly275	Gly275Ser (G275S)	[13]
		Tyr277	Tyr277Cys (Y277C)	[19]
		Gly279	Gly279Cys, Gly279Val (G279C, G279V)	[16, 17, 20, 21]
		Arg288	Arg288Met (R288M)	[19]

Table A An alignment of (potentially) important amino acid residues that are either reported in literature (the third column), or identified by computational analysis (salt bridge analysis, hydrogen bond analysis and solvent accessible surface area analysis) of the SMN-related structures determined by solution-state NMR spectroscopy (the first column) or X-ray crystallography (the second column). The fourth column provides the SMA patient-identified point mutation(s) for different residues, the fifth column contains the reference(s) for the mutation(s). In the computational analysis, an amino acid residue is considered as potentially important only if its side chain is involved in a salt bridge or a hydrogen bond, or its SASA is smaller that 30% of its standard SASA value.

1.1.4 Chemical shift analysis of the SMN-related NMR structures

In the experimantal structure determination by NMR spectroscopy, missing chemical shift assignment is one major factor that allows high degree of geometric freedom, especially for the side chain structures, and consequently high degree of freedom in the surface charge distribution, resulting in inadequate accuracy in the geometric definition of the NMR structures. As a result, an analysis was carried out to examine the chemical shift assignments of the four NMR structures (with PDB IDs 1g5v, 2leh, 4a4e and 4a4g, respectively.) deposited in the BMRB database [22]. A list of the missing assignments is provided in the following Table C.

Item	PDB ID	BMRB ID	Title of the NMR-determined structure
1	1g5v [23]	4899	Solution structure of the TUDOR domain of the human SMN protein
2	2leh [1]	17711	Solution structure of the core SMN-GEMIN2 complex
3	4a4e [24]	NA	Solution structure of SMN TUDOR domain in complex with symmetrically dimethylated arginine
4	4a4g [24]	NA	Solution structure of SMN TUDOR domain in complex with asymmetrically dimethylated arginine
5	5mf9 [25]	34067	Solution structure of the RBM5 OCRE domain in complex with polyproline SMN peptide

Table B Protein Data Bank (PDB) [26] and BioMagResBank (BMRB) [22] IDs and titles of the five NMR-determined structures (as of January 25, 2017). Among them, 5mf9 was excluded as there is no alignment between the 5mf9 chain B sequence (the one that belongs to SMN) and the real amino acid sequence of SMN. In this table, NA means that the BMRB ID is not available for the specified PDB structure.

**1g5v**	B + S	B	S	**2leh**	B + S	B	S
Expected	773	280	493	Expected	3062	1075	1987
Observed	616	224	393	Observed	2544	1027	1517
Missing	158	58	100	Missing	518	48	470
Ratio (missing)	20.43%	20.71%	20.28%	Ratio (missing)	16.91%	4.46%	23.65%
**4a4e**	B + S	B	S	**4a4g**	B + S	B	S
Expected	865	325	540	Expected	865	325	540
Observed	716	254	462	Observed	715	253	462
Missing	149	71	78	Missing	150	72	78
Ratio (missing)	17.22%	21.84%	14.44%	Ratio (missing)	17.34%	22.15%	14.44%

Table C ^{1}H, ^{15}N and ^{13}C nuclei with missing chemical shift assignments for both backbone and side chains in the four NMR structures. In this table, Expected represents the number of ^{1}H, ^{15}N and ^{13}C nuclei from the the four NMR structures structure which should be observable by NMR spectroscopy, whereas Observed means the number of ^{1}H, ^{15}N and ^{13}C nuclei from the the four NMR structures structure that are observed in NMR spectroscopy and deposited in the BMRB database. B and S reresent backbone and side chain, respectively. 1g5v, 2leh, 4a4e and 4a4g represent the PDB IDs of the four NMR structures, as listed in Table B.

Residue	Number	Nucleus or nuclei whose chemical shift(s) is/are missing
ILE113	1	C
GLN136	3	C, HG3, CD
GLY131	1	C
LYS119	7	C, HD3, HE3, HZ2, NZ, HZ1, HZ3
LYS93	7	C, HD3, HE3, HZ2, NZ, HZ1, HZ3
GLN90	3	C, HG3, CD
LYS97	7	C, HD3, HE3, HZ2, NZ, HZ1, HZ3
SER139	2	C, HG
TRP102	4	CD2, C, CG, CE2
THR112	2	C, HG1
TYR130	8	C, HE2, CG, CZ, HH, HD2, CD2, CE2
ILE116	1	C
CYS107	2	C, HG
SER143	2	C, HG
THR128	2	C, HG1
SER115	2	C, HG
SER99	2	C, HG
LEU138	1	C
GLU134	2	C, CD
GLU135	2	C, CD
VAL126	1	C
ARG133	11	HG3, HE, NE, HH22, HH21, C, CZ, NH1, NH2, HH12, HH11
VAL124	1	C
VAL125	1	C
THR122	2	C, HG1
ILE101	2	C, HG13
ASP117	2	C, CG
ALA114	1	C
ALA111	1	C
ASN137	5	C, HD22, HD21, CG, ND2
ASN132	2	C, CG
ILE145	1	C
PHE118	6	C, HE2, CG, HD2, CD2, CE2
GLY129	1	C
PRO144	3	C, HD3, N
CYS98	2	C, HG
LEU141	1	C
TYR127	8	C, HE2, CG, CZ, HH, HD2, CD2, CE2
TRP92	4	CD2, C, CG, CE2
ASP140	2	C, CG
GLN91	2	C, CD
ILE108	1	C

TYR109	8	C, HE2, CG, CZ, HH, HD2, CD2, CE2
LEU142	1	C
CYS123	2	C, HG
GLY95	1	C
SER103	2	C, HG
GLY106	1	C
GLU104	2	C, CD
ALA100	1	C
ARG120	10	HE, NE, HH22, HH21, C, CZ, NH1, NH2, HH12, HH11
GLU121	2	C, CD
VAL94	1	C
ASP96	2	C, CG
ASP105	2	C, CG
PRO110	2	C, N

Table D Missing chemical shift assignments for the NMR structure of 1g5v [23] (the PDB ID of the NMR structure, as listed in Table B). This table was prepared according to the chemical shifts deposited in BMRB database [22] for the Cp5 NMR structure(PDB [26] access code: 1g5v). In the second column, Number represents the number of nucleus/nuclei whose assignment(s) is/are missing in the BMRB database for each residue. The backgrounds of the rows for charged residues are colored gray.

Residue	Number	Nucleus or nuclei whose chemical shift(s) is/are missing
GLY95	5	C, H2, H3, H1, N
TYR96	5	CG, N, CZ, HH, H
SER97	1	HG
PRO98	1	N
THR99	1	HG1
LEU100	0	
GLN101	4	CD, HE22, HE21, NE2
TRP102	11	HH2, CZ2, CZ3, CD2, HE3, CH2, HZ3, HZ2, CG, CE3, CE2
GLN103	4	CD, HE22, HE21, NE2
GLN104	1	CD
GLN105	4	CD, HE22, HE21, NE2
GLN106	1	CD
VAL107	0	
ALA108	0	
GLN109	4	CD, HE22, HE21, NE2
PHE110	7	HZ, HE2, CE1, CG, CZ, HE1, CE2
SER111	1	HG
THR112	1	HG1
VAL113	0	
ARG114	9	HE, NE, HH22, HH21, CZ, NH1, NH2, HH12, HH11
GLN115	4	CD, HE22, HE21, NE2
ASN116	4	HD22, HD21, CG, ND2
VAL117	0	
ASN118	4	HD22, HD21, CG, ND2
LYS119	4	HZ2, NZ, HZ1, HZ3
HIS120	4	HE2, CG, ND1, NE2
ARG121	9	HE, NE, HH22, HH21, CZ, NH1, NH2, HH12, HH11
SER122	1	HG
HIS123	5	HE2, CG, CD2, ND1, NE2
TRP124	7	CZ3, CD2, HE3, HZ3, CG, CE3, CE2
LYS125	4	HZ2, NZ, HZ1, HZ3
SER126	1	HG
GLN127	4	CD, HE22, HE21, NE2
GLN128	4	CD, HE22, HE21, NE2
LEU129	0	
ASP130	1	CG
SER131	1	HG
ASN132	4	HD22, HD21, CG, ND2
VAL133	0	
THR134	1	HG1
MET135	0	
PRO136	1	N

LYS137	4	HZ2, NZ, HZ1, HZ3
SER138	1	HG
GLU139	1	CD
ASP140	1	CG
GLU141	1	CD
GLU142	1	CD
GLY143	0	
TRP144	6	CZ3, CD2, HE3, CG, CE3, CE2
LYS145	10	HD3, HD2, HE2, HE3, HZ2, CE, CD, NZ, HZ1, HZ3
LYS146	4	HZ2, NZ, HZ1, HZ3
PHE147	3	HZ, CG, CZ
CYS148	1	HG
LEU149	0	
GLY150	0	
GLU151	7	C, HA, CB, CA, CD, HB3, HB2
LYS152	8	HD3, HD2, HZ2, NZ, HZ1, HZ3, H, N
LEU153	0	
CYS154	1	HG
ALA155	0	
ASP156	1	CG
GLY157	0	
ALA158	0	
VAL159	0	
GLY160	0	
PRO161	1	N
ALA162	0	
THR163	1	HG1
ASN164	4	HD22, HD21, CG, ND2
GLU165	1	CD
SER166	1	HG
PRO167	1	N
GLY168	0	
ILE169	0	
ASP170	1	CG
TYR171	3	CG, CZ, HH
VAL172	1	C
GLN173	5	C, CD, HE22, HE21, NE2
ILE174	0	
GLY175	0	
PHE176	4	HZ, CA, CG, CZ
PRO177	7	C, HA, CB, CA, N, HB3, HB2
PRO178	4	HA, N, HB3, HB2
LEU179	0	

LEU180	1	C
SER181	4	N, H, HA, HG
ILE182	0	
VAL183	0	
SER184	1	HG
ARG185	10	HE, NE, HH22, HH21, CD, CZ, NH1, NH2, HH12, HH11
MET186	0	
ASN187	4	HD22, HD21, CG, ND2
GLN188	4	CD, HE22, HE21, NE2
ALA189	0	
THR190	1	HG1
VAL191	0	
THR192	1	HG1
SER193	1	HG
VAL194	0	
LEU195	0	
GLU196	1	CD
TYR197	3	CG, CZ, HH
LEU198	0	
SER199	1	HG
ASN200	4	HD22, HD21, CG, ND2
TRP201	13	HH2, CZ2, CZ3, CD1, CD2, HE3, CH2, HZ3, HZ2, CG, CE3, CE2, HD1
PHE202	11	HZ, HE2, CE1, HD1, CG, CZ, HD2, CD1, CD2, HE1, CE2
GLY203	0	
GLU204	1	CD
ARG205	9	HE, NE, HH22, HH21, CZ, NH1, NH2, HH12, HH11
ASP206	1	CG
PHE207	1	CG
THR208	1	HG1
PRO209	1	N
GLU210	1	CD
LEU211	0	
GLY212	0	
ARG213	9	HE, NE, HH22, HH21, CZ, NH1, NH2, HH12, HH11
TRP214	4	CD2, CH2, CG, CE2
LEU215	2	CG, HG
TYR216	3	CG, CZ, HH
ALA217	0	
LEU218	0	
LEU219	0	
ALA220	0	
CYS221	1	HG
LEU222	0	

GLU223	1	CD
LYS224	8	HD3, HD2, HZ2, CE, CD, NZ, HZ1, HZ3
PRO225	1	N
LEU226	0	
LEU227	0	
PRO228	1	N
GLU229	1	CD
ALA230	0	
HIS231	4	HE2, CG, ND1, NE2
SER232	1	HG
LEU233	0	
ILE234	0	
ARG235	18	HG3, HE, HG2, NE, HH22, HH21, CB, CG, CD, CZ, NH1, NH2, HD3, HD2, HH12, HH11, HB3, HB2
GLN236	4	CD, HE22, HE21, NE2
LEU237	0	
ALA238	0	
ARG239	9	HE, NE, HH22, HH21, CZ, NH1, NH2, HH12, HH11
ARG240	19	HG3, HA, HE, HG2, NE, HH22, HH21, CB, CG, CD, CZ, NH1, NH2, HD3, HD2, HH12, HH11, HB3, HB2
CYS241	1	HG
SER242	2	C, HG
GLU243	1	CD
VAL244	0	
ARG245	9	HE, NE, HH22, HH21, CZ, NH1, NH2, HH12, HH11
LEU246	0	
LEU247	0	
VAL248	0	
ASP249	1	CG
SER250	1	HG
LYS251	4	HZ2, NZ, HZ1, HZ3
ASP252	1	CG
ASP253	2	C, CG
GLU254	5	C, HA, CD, HB3, HB2
ARG255	18	HG3, HE, HG2, NE, HH22, HH21, CB, CG, CD, CZ, NH1, NH2, HD3, HD2, HH12, HH11, HB3, HB2
VAL256	1	C
PRO257	1	N
ALA258	0	
LEU259	0	
ASN260	4	HD22, HD21, CG, ND2
LEU261	4	CG, HG, HB3, HB2
LEU262	0	

ILE263	0	
CYS264	1	HG
LEU265	0	
VAL266	1	C
SER267	1	HG
ARG268	9	HE, NE, HH22, HH21, CZ, NH1, NH2, HH12, HH11
TYR269	3	CG, CZ, HH
PHE270	7	HZ, HE2, CE1, CG, CZ, HE1, CE2
ASP271	1	CG
GLN272	7	HG2, CG, HG3, CD, HE22, HE21, NE2
ARG273	9	HE, NE, HH22, HH21, CZ, NH1, NH2, HH12, HH11
ASP274	1	CG
LEU275	0	
ALA276	0	
ASP277	1	CG
GLU278	1	CD
PRO279	1	N
SER280	1	HG
LEU281	0	
GLU282	1	CD
TYR283	3	CG, CZ, HH
GLY26	8	C, H2, H3, CA, H1, N, HA2, HA3
GLN27	3	CD, H, N
SER28	1	HG
ASP29	1	CG
ASP30	1	CG
SER31	1	HG
ASP32	1	CG
ILE33	0	
TRP34	4	CD2, C, CG, CE2
ASP35	5	C, CB, CG, HB3, HB2
ASP36	4	CG, N, H, HA
THR37	1	HG1
ALA38	0	
LEU39	0	
ILE40	0	
LYS41	4	HZ2, NZ, HZ1, HZ3
ALA42	0	
TYR43	2	CG, CZ
ASP44	1	CG
LYS45	4	HZ2, NZ, HZ1, HZ3
ALA46	0	
VAL47	0	

ALA48	0	
SER49	1	HG
PHE50	1	CG
LYS51	4	HZ2, NZ, HZ1, HZ3

Table E Missing chemical shift assignments for the NMR structure of 2leh [1](the PDB ID of the NMR structure, as listed in Table B). This table was prepared according to the chemical shifts deposited in BMRB database [22] for the Cp5 NMR structure(PDB [26] access code: 2leh). In the second column, Number represents the number of nucleus/nuclei whose assignment(s) is/are missing in the BMRB database for each residue. The backgrounds of the rows for charged residues are colored gray.

Residue	Number	Nucleus or nuclei whose chemical shift(s) is/are missing
ILE113	1	C
2MR1148	0	
VAL125	1	C
GLY131	1	C
LYS119	8	C, HE2, HE3, HZ2, CE, NZ, HZ1, HZ3
LYS93	5	C, HZ2, NZ, HZ1, HZ3
ILE108	1	C
LYS97	5	C, HZ2, NZ, HZ1, HZ3
GLN136	2	C, CD
SER139	2	C, HG
TRP102	4	CD2, C, CG, CE2
THR112	2	C, HG1
TYR130	4	C, CG, CZ, HH
ILE116	1	C
TYR109	4	C, CG, CZ, HH
SER143	2	C, HG
THR128	2	C, HG1
SER115	2	C, HG
SER99	2	C, HG
LEU138	1	C
GLU134	2	C, CD
ALA87	1	C
VAL126	1	C
ARG133	10	HE, NE, HH22, HH21, C, CZ, NH1, NH2, HH12, HH11
VAL124	1	C
PHE118	2	C, CG
THR122	2	C, HG1
LEU89	1	C
ILE101	1	C
ASN84	6	C, H2, H1, CG, N, H3
ALA114	1	C
ALA111	1	C
ASN137	2	C, CG
ASN132	3	C, CA, CG
CYS146	2	C, HG
THR85	2	C, HG1
ALA86	2	C, CA
GLY129	1	C
ASP117	3	C, CA, CG
PRO144	2	C, N
CYS98	1	C
LEU141	1	C

TYR127	3	C, CG, CZ
TRP92	4	CD2, C, CG, CE2
ASP140	2	C, CG
ILE145	1	C
GLN91	2	C, CD
GLN90	2	C, CD
CYS107	2	C, HG
LEU142	1	C
CYS123	1	C
GLU135	2	C, CD
GLY95	1	C
SER103	2	C, HG
GLY106	1	C
GLU104	2	C, CD
SER88	2	C, HG
ARG120	10	HE, NE, HH22, HH21, C, CZ, NH1, NH2, HH12, HH11
GLU147	2	C, CD
GLU121	3	C, CD, N
PRO110	2	C, N
VAL94	1	C
ASP96	2	C, CG
ASP105	2	C, CG
ALA100	1	C

Table F Missing chemical shift assignments for the NMR structure of 4a4e [24](the PDB ID of the NMR structure, as listed in Table B). This table was prepared according to the chemical shifts deposited in BMRB database [22] for the Cp5 NMR structure(PDB [26] access code: 4a4e). In the second column, Number represents the number of nucleus/nuclei whose assignment(s) is/are missing in the BMRB database for each residue. The backgrounds of the rows for charged residues are colored gray.

Residue	Number	Nucleus or nuclei whose chemical shift(s) is/are missing
ILE113	1	C
VAL125	1	C
GLY131	1	C
LYS119	8	C, HE2, HE3, HZ2, CE, NZ, HZ1, HZ3
LYS93	5	C, HZ2, NZ, HZ1, HZ3
ILE108	1	C
LYS97	5	C, HZ2, NZ, HZ1, HZ3
GLN136	2	C, CD
DA21148	0	
SER139	2	C, HG
TRP102	4	CD2, C, CG, CE2
THR112	2	C, HG1
TYR130	4	C, CG, CZ, HH
ILE116	1	C
TYR109	4	C, CG, CZ, HH
SER143	2	C, HG
THR128	2	C, HG1
SER115	2	C, HG
SER99	2	C, HG
LEU138	1	C
GLU134	2	C, CD
ALA87	1	C
VAL126	1	C
ARG133	10	HE, NE, HH22, HH21, C, CZ, NH1, NH2, HH12, HH11
VAL124	1	C
PHE118	2	C, CG
THR122	2	C, HG1
LEU89	1	C
ILE101	1	C
ASN84	6	C, H2, H1, CG, N, H3
ALA114	1	C
ALA111	1	C
ASN137	2	C, CG
ASN132	2	C, CG
CYS146	2	C, HG
THR85	2	C, HG1
ALA86	3	C, CA, HA
GLY129	3	C, H, N
ASP117	3	C, CA, CG
PRO144	2	C, N
CYS98	1	C
LEU141	1	C

TYR127	3	C, CG, CZ
TRP92	4	CD2, C, CG, CE2
ASP140	2	C, CG
ILE145	1	C
GLN91	2	C, CD
GLN90	2	C, CD
CYS107	2	C, HG
LEU142	1	C
CYS123	1	C
GLU135	2	C, CD
GLY95	1	C
SER103	2	C, HG
GLY106	1	C
GLU104	2	C, CD
SER88	2	C, HG
ARG120	10	HE, NE, HH22, HH21, C, CZ, NH1, NH2, HH12, HH11
GLU147	2	C, CD
GLU121	2	C, CD
PRO110	2	C, N
VAL94	1	C
ASP96	2	C, CG
ASP105	2	C, CG
ALA100	1	C

Table G Missing chemical shift assignments for the NMR structure of 4a4g [24](the PDB ID of the NMR structure, as listed in Table B). This table was prepared according to the chemical shifts deposited in BMRB database [22] for the Cp5 NMR structure(PDB [26] access code: 4a4g). In the second column, Number represents the number of nucleus/nuclei whose assignment(s) is/are missing in the BMRB database for each residue. The backgrounds of the rows for charged residues are colored gray.

References

1 Kathryn L. Sarachan, Kathleen G. Valentine, Kushol Gupta, Veronica R. Moorman, John M. Gledhill, Matthew Bernens, Cecilia Tommos, A. Joshua Wand, and Gregory D. Van Duyne. Solution structure of the core SMN-Gemin2 complex. *Biochemical Journal*, 445(3):361–370, 2012.

2 U. R. Monani, M. T. Pastore, T. O. Gavrilina, S Jablonka, T. T. Le, C Andreassi, J. M. Dicocco, C Lorson, E. J. Androphy, and M Sendtner. A transgene carrying an A2G missense mutation in the SMN gene modulates phenotypic severity in mice with severe (type I) spinal muscular atrophy. *Journal of Cell Biology*, 160(1):41–52, 2003.

3 Brunhilde Wirth. An update of the mutation spectrum of the survival motor neuron gene *SMN1* in autosomal recessive spinal muscular atrophy (sma). *Human Mutation*, 15(3): 228–237, 2000.

4 Y. Sun, M Grimmler, V Schwarzer, F Schoenen, U Fischer, and B Wirth. Molecular and functional analysis of intragenic SMN1 mutations in patients with spinal muscular atrophy. *Human Mutation*, 25(1):64–71, 2005.

5 T Kotani, R Sutomo, T. H. Sasongko, A. H. Sadewa, Gunadi, T Minato, E Fujii, S Endo, M. J. Lee, and H Ayaki. A novel mutation at the n-terminal of SMN Tudor domain inhibits its interaction with target proteins. *Journal of Neurology*, 254(5):624–630, 2007.

6 Olivier Clermont, Philippe Burlet, Paule Benit, Dominique Chanterau, Pascale Saugier Veber, Arnold Munnich, and Veronica Cusin. Molecular analysis of sma patients without homozygous smn1 deletions using a new strategy for identification of smn1 subtle mutations. *Human Mutation*, 24(5):417–27, 2004.

7 T. W. Prior. Spinal muscular atrophy diagnostics. *Journal of Child Neurology*, 22(8): 952–956, 2007.

8 V Sossi, A Giuli, T Vitali, F Tiziano, M Mirabella, A Antonelli, G Neri, and C Brahe. Premature termination mutations in exon 3 of the smn1 gene are associated with exon skipping and a relatively mild sma phenotype. *European Journal of Human Genetics Ejhg*, 9(2):113–120, 2001.

9 E Workman, L Saieva, T. L. Carrel, T. O. Crawford, D. Liu, C Lutz, C. E. Beattie, L Pellizzoni, and A. H. Burghes. A SMN missense mutation complements SMN2 restoring snRNPs and rescuing SMA mice. *Human Molecular Genetics*, 18(12):2215–2229, 2009.

10 I Cuscó, M. J. Barceló, Río E Del, M Baiget, and E. F. Tizzano. Detection of novel mutations in the SMN Tudor domain in type I SMA patients. *Neurology*, 63(1):146–149, 2004.

11 Lars Brichta, Lutz Garbes, and Maria Jedrzejowska. Nonsense-mediated messenger rna decay of survival motor neuron 1 causes spinal muscular atrophy. *Human Genetics*, 123(2):141–153, 2008.

12 Eva Zapletalová, Petra Hedvičáková, Libor Kozák, Petr Vondráček, Renata Gaillyová, Tat'ána Mařı´ková, Zdeněk Kalina, Věra Jüttnerová, Jiřı´ Fajkus, and Lenka Fajkusová. Analysis of point mutations in the SMN1 gene in SMA patients bearing a single SMN1 copy. *Neuromuscular Disorders*, 17(6):476–481, 2007.

13 Laura Alías, Sara Bernal, Pablo Fuentes-Prior, María Jesus Barceló, Eva Also, Rebeca Martínez-Hernández, Francisco J. Rodríguez-Alvarez, Yolanda Martín, Elena Aller, Elena Grau, Ana Peciña, Guillermo Antiñolo, Enrique Galán, Alberto L. Rosa, Miguel Fernández-Burriel, Salud Borrego, José M. Millán, Concepción Hernández-Chico, Montserrat Baiget, and Eduardo F. Tizzano. Mutation update of spinal muscular atrophy in spain: molecular characterization of 745 unrelated patients and identification of four novel mutations in the SMN1 gene. *Human Genetics*, 125(1):29–39, 2008.

14 C Tsai, Y Jong, C Hu, C Chen, M Shih, C Chang, and J Chang. Molecular analysis of smn, naip and p44 genes of sma patients and their families. *Journal of the Neurological Sciences*, 190(2):35–40, 2001.

15 C. F. Rochette, L. C. Surh, P. N. Ray, P. E. Mcandrew, T. W. Prior, A. H. M. Burghes, M. Vanasse, and L. R. Simard. Molecular diagnosis of non-deletion sma patients using quantitative pcr of smn exon 7. *neurogenetics*, 1(2):141–147, 1997.

16 C. L. Lorson, J Strasswimmer, J. M. Yao, J. D. Baleja, E Hahnen, B Wirth, T. Le, A. H. Burghes, and E. J. Androphy. Smn oligomerization defect correlates with spinal muscular atrophy severity. *Nature Genetics*, 19(1):63–6, 1998.

17 Karl B. Shpargel and Joseph G. Gall. Gemin proteins are required for efficient assembly of Sm-class ribonucleoproteins. *Proceedings of the National Academy of Sciences of the United States of America*, 102(48):17372–73717, 2005.

18 E Hahnen, J Schönling, S Rudnik-Schöneborn, H Raschke, K Zerres, and B Wirth. Missense mutations in exon 6 of the survival motor neuron gene in patients with spinal muscular atrophy (SMA). *Human Molecular Genetics*, 6(5):821–285, 1997.

19 Qu Yu-jin, Du Juan, Li Er-zhen, Bai Jin-li, Jin Yu-wei, Wang Hong, and Song Fang. Subtle mutations in the SMN1 gene in chinese patients with SMA: p.arg288met mutation causing SMN1 transcript exclusion of exon7. *BMC Medical Genetics*, 13(1), sep 2012.

20 Kevin Talbot, Christopher P. Ponting, Aspasia M. Theodosiou, Nanda R. Rodrigues, Robert Surtees, Roger Mountford, and Kay E. Davies. Missense mutation clustering in the survival motor neuron gene: a role for a conserved tyrosine and glycine rich region of the protein in RNA metabolism? *Human Molecular Genetics*, 6(3):497–500, 1997.

21 C. H. Wang, B. D. Papendick, P Bruinsma, and J. K. Day. Identification of a novel missense mutation of the SMN^T gene in two siblings with spinal muscular atrophy. *neurogenetics*, 1(4):273–276, 1998.

22 J. L. Markley, E. L. Ulrich, H. M. Berman, K Henrick, H Nakamura, and H Akutsu. Biomagresbank (BMRB) as a partner in the worldwide Protein Data Bank (wwPDB): new policies affecting biomolecular NMR depositions. *Journal of Biomolecular NMR*, 40 (3):153–155, 2008.

23 Michael Sattler, Philipp Selenko, Remco Sprangers, Gunter Stier, Dirk Bühler, and Utz Fischer. SMN Tudor domain structure and its interaction with the Sm proteins. *Nature Structural Biology*, 8(1):27–31, 2001.

24 K Tripsianes, T Madl, M Machyna, D Fessas, C Englbrecht, U Fischer, K. M. Neugebauer, and M Sattler. Structural basis for dimethylarginine recognition by the Tudor domains of human SMN and spf30 proteins. *Nature Structural & Molecular Biology*, 18 (12):1414–1420, 2011.

25 A Mourao, S Bonnal, K Soni, L Warner, R Bordonne, J Valcarcel, and M Sattler. Structural basis for the recognition of spliceosomal Smn/b/b' proteins by the rbm5 ocre domain in splicing regulation. *Elife*, 5, 2016.

26 H Berman, K Henrick, and H Nakamura. Announcing the worldwide Protein Data Bank. *Nature Structural Biology*, 10(12):980, 2003.
